# The Effect of Different Photoperiods in Circadian Rhythms of *Per3* Knockout Mice

**DOI:** 10.1155/2014/170795

**Published:** 2014-05-08

**Authors:** D. S. Pereira, D. R. van der Veen, B. S. B. Gonçalves, S. Tufik, M. von Schantz, S. N. Archer, M. Pedrazzoli

**Affiliations:** ^1^Departamento de Psicobiologia, Universidade Federal de São Paulo (UNIFESP), Rua Napoleão de Barros 925, 3*º* Andar, 04024-002 São Paulo, SP, Brazil; ^2^Faculty of Health and Medical Science, University of Surrey, Guildford, UK; ^3^Departamento de Fisiologia, Universidade Federal do Rio Grande do Norte (UFRN), Natal, RN, Brazil; ^4^Instituto Federal Sudeste de Minas Gerais, Campus Barbacena, Barbacena, MG, Brazil; ^5^Escola de Artes, Ciências e Humanidades, Universidade de São Paulo (USP), São Paulo, SP, Brazil

## Abstract

The aim of this study was to analyse the circadian behavioural responses of mice carrying a functional knockout of the *Per3* gene (*Per*3^−/−^) to different light : dark (L : D) cycles. Male adult wild-type (WT) and *Per*3^−/−^ mice were kept under 12-hour light : 12-hour dark conditions (12L : 12D) and then transferred to either a short or long photoperiod and subsequently released into total darkness. All mice were exposed to both conditions, and behavioural activity data were acquired through running wheel activity and analysed for circadian characteristics during these conditions. We observed that, during the transition from 12L : 12D to 16L : 8D, *Per*3^−/−^ mice take approximately one additional day to synchronise to the new L : D cycle compared to WT mice. Under these long photoperiod conditions, *Per*3^−/−^ mice were more active in the light phase. Our results suggest that *Per*3^−/−^ mice are less sensitive to light. The data presented here provides further evidence that *Per3* is involved in the suppression of behavioural activity in direct response to light.

## 1. Introduction

Circadian (~24-hour) rhythms are driven by internal clocks, which are entrained by external timing cues (Zeitgebers). One of the most important Zeitgebers is the environmental light : dark (L : D) cycle. Photic signals entrain the circadian clock in the suprachiasmatic nuclei (SCN) of the hypothalamus, and the entrained signal is distributed to the hierarchical network of clocks in peripheral tissues [1].

In the last four decades, substantial progress has been made in the understanding of the molecular basis of circadian rhythmicity. Several clock genes have been identified in mammals (for review, [2]). Studies using functional clock gene knockout mice have shown alterations in the endogenous circadian period length, loss of persistence of circadian rhythms, and disturbed sleep-wake cycles [3, 4].

Among the so-called clock genes, reports on the function of* Per3* have been the most inconclusive. While studies in animals suggest that the* Per3* gene is not critical for regulating circadian rhythms based on the small changes found in the free-running period and the lack of differential responses to light pulses in functional knockout animals [3, 4], human studies have shown that this gene is strongly associated with chronotypes, circadian dysfunction, and the homeostatic regulation of sleep [5–9]. More recently, the absence of* Per3* has been shown to differentially affect peripheral oscillators [10].

Our understanding of the function of* Per3* was improved by the finding that functional* Per3 *knockout mice (*Per3* ^−*/*−^) are characterised by altered sensitivity to light [11] and altered sleep homeostatic responses to sleep deprivation [9]. It has also been proposed that, in humans, the* PER3* gene could be involved in the entrainment to differential seasonal light signals created by latitude [7, 12]. Based on the previous finding that the* Per3* gene plays a role in circadian light sensitivity, the aim of this study was to analyse whether* Per3* ^−*/*−^ mice respond differently to different photoperiods.

## 2. Materials and Methods

### 2.1. Animals

C57BL/6* Per3* ^−*/*−^ mice were generated as previously described [4, 11]. Six wild-type (WT) mice and six* mPer3* ^−*/*−^ mice, originating from heterozygous backcrosses on a C57Bl/6 background and 3 months of age, were housed in running wheel cages in light-tight, sound-attenuated cabinets, and activity was recorded in 1 min bins (Clocklab, Actimetrics, Wilmette, IL). The light intensity was 800 +/− 13 mW/m^2^ (mean +/− SEM) in the light phase. The temperature was maintained at 19 to 22°C and relative humidity at 55% ± 10%. The animals were provided with food (transgenic mouse diet, B & K Universal Ltd, Hull, UK) and water* ad libitum.*

The experiments had previously received a favourable opinion from the University of Surrey Animal Ethics Committee and were carried out under UK Home Office License in accordance with the Declaration of Helsinki.

### 2.2. Light Entrainment

**Figure 1:**
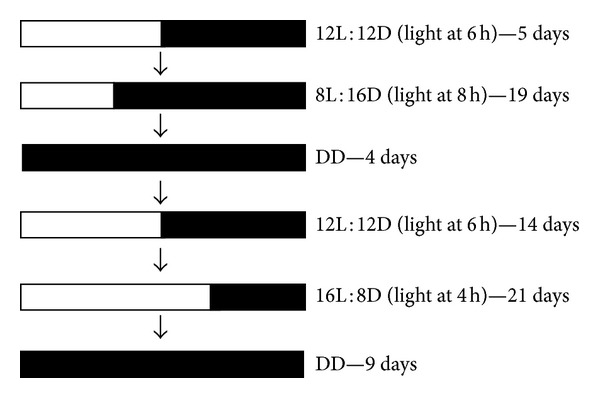
Schematic representation of the experimental protocol. All mice were submitted to this protocol in the same sequence. L : D = light : dark, DD = dark : dark.

Mice were entrained to 12L : 12D for 5 days and then exposed to the following sequential L : D schemes: 8L : 16D for 19 days, 4 days in constant dark (DD), 12L : 12D for 14 days, 16L : 8D for 22 days, and 9 days in DD (Figure 1).

### 2.3. Behavioural and Statistic Analysis

Behavioural (periodogram) analysis [13]) and graphical output (actograms) were produced using the* El Temps* software (A. Díez-Noguera, University of Barcelona, 1999), and statistical significance was tested using Statistica software (StatSoft Inc., 1984–2007, Tulsa, OK). The Kolmogorov-Smirnov test was used to test for normality of distribution. Normally distributed data were compared between groups using Student's *t* test, and data that were not normally distributed were compared using the Mann-Whitney test. The significance level was set at *P* < 0.05.

The phase angle of entrainment was calculated under all light conditions and defined as the difference in minutes between the onset of darkness and the activity onset. Positive phase angles indicate that the animal became active after lights off, and negative phase angles indicate that the animal became active before the light was turned off. For behavioural phase determination, we smoothed the data using a boxcar smoothing approach with a 2-hour window. For each day, we determined for the first instance that the activity level of the smoothed activity exceeded (onset) the 24-hour average. To filter out any fluctuations (e.g., the typical late night “dip” in behavioural activity observed in C57Bl/6 mice), we set an additional requirement that any onset was valid only when the activity in the 2 hours preceding this onset was lower than 10 running wheel revolutions and at least 50 revolutions in the 2 hours after the onset.

We visually determined the number of days (transients) required to resynchronise after L : D cycle change for each animal. We considered that transients were fully completed at the new L : D condition when activity onset stabilised at a time point and remained at the same point at least for two consecutive days.

The amount of running wheel activity during the light and dark phase was expressed in centimeters (2*πR* of the running wheel, where *R* is the radius of the wheel); total amount of activity per animal per L : D schedule was calculated and then averaged by group. Transients of light transitions were excluded in each light or dark phase to calculate the amount of running wheel activity.

## 3. Results

Periodogram analysis (all individual periodograms are included in the supplementary material) showed that mice of both genotypes were entrained to a near 24 h period (no significant difference in period length).

**Figure 2:**
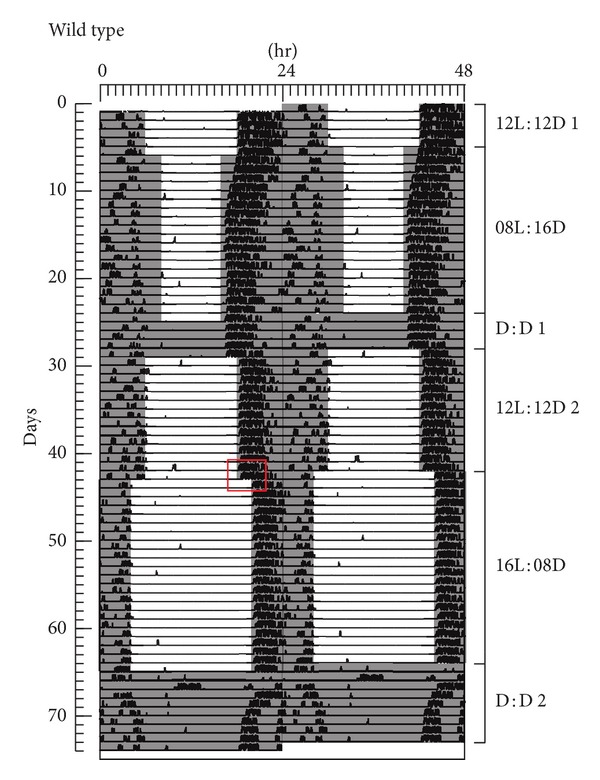
A representative double-plot actogram of one WT mouse. Mice were entrained to the following light : dark conditions: 12L : 12D for 5 days; 8L : 16D for 19 days; constant dark (DD) for 4 days; 12L : 12D for 14 days; 16L : 8D for 22 days and DD for 9 days. The red rectangle depicts the transition from 12L : 12D to 16L : 8D.

**Figure 3:**
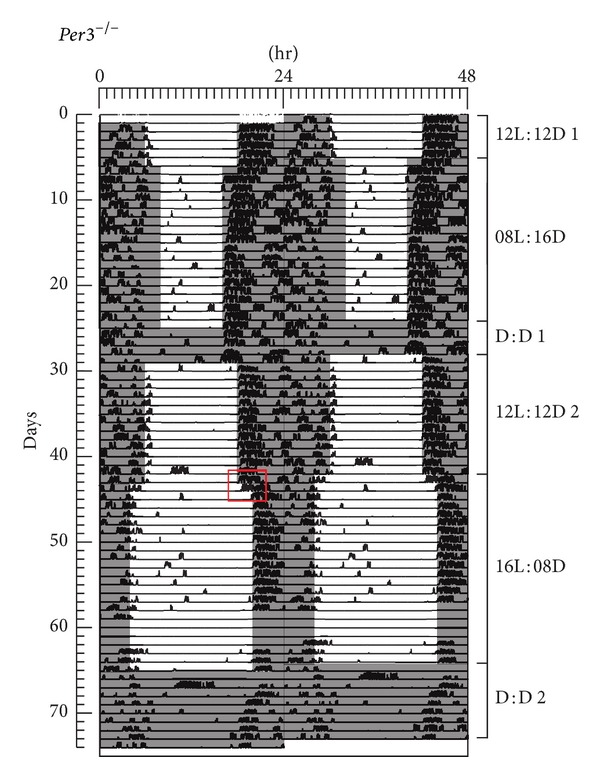
A representative double-plot actogram of one* mPer3* ^−*/*−^ mouse. Mice were entrained to the following light : dark conditions : 12L : 12D for 5 days; 8L : 16D for 19 days; constant dark (DD) for 4 days; 12L : 12D for 14 days; 16L : 8D for 22 days and DD for 9 days. The red rectangle depicts the transition from 12L : 12D to 16L : 8D.

Figures 2 and 3 show representative examples of activity plots for a WT (Animal 8 in Supplementary Material available online at http://dx.doi.org/10.1155/2014/170795) and* Per3* ^−*/*−^ (Animal 2 in Supplementary Material) animal, respectively. In the transition from 12L : 12D to 8L : 16D, we observed no difference between WT and* Per3* ^−*/*−^ mice in terms of behavioural reentrainment. The average transient for both groups was 5.5 days (±1.6 and ±2.9 SD, WT and* Per3* ^−*/*−^, resp.). By contrast, for the transition from 12L : 12D to 16L : 8D,* Per3* ^−*/*−^ mice took on average 2.0 ± 0.8 (±SD) days to synchronise while WT mice responded rapidly to the new light stimulus (*Z*_adjusted_ = −2.3, *P* = 0.02). Mice quickly synchronised to the long photoperiod, suggesting that behavioural activity in what is now the light phase is, in fact, predominantly masked by light. When mice were released to DD after 16L : 8D, they did not retain activity patterns seen in 16L : 8D, but, after a few transients, they seemed to return to the same phase where they were in the previous 12L : 12D cycles.

**Figure 4:**
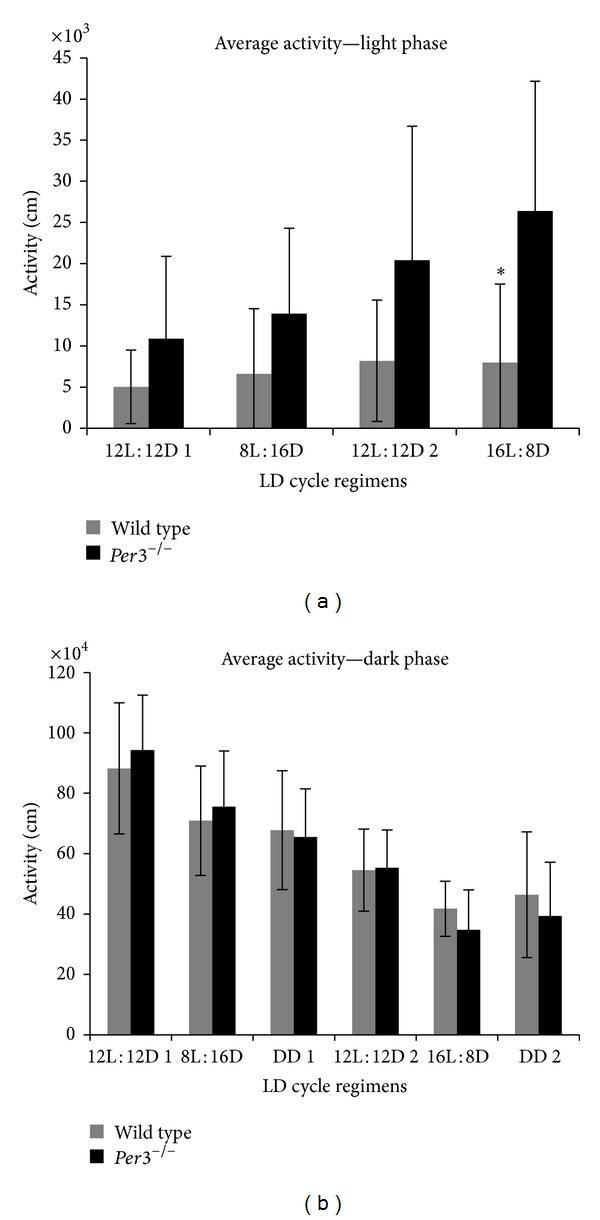
Total activity in light (a) and dark (b) phases. Values are represented as mean ± SD. Gray bars represent wild-type mice, and black bars show* mPer3* ^−*/*−^ mice. *Statistically significant *P* < 0.05.

In agreement with previous data [9],* Per3* ^−*/*−^ mice were overall more active than WT mice. Although increased activity can be observed in the light phase during all L : D conditions, this increase reached statistical significance during the longer photoperiod cycles (*Z*_adjusted_ = −2.24, *P* = 0.02, Figure 4).

**Figure 5:**
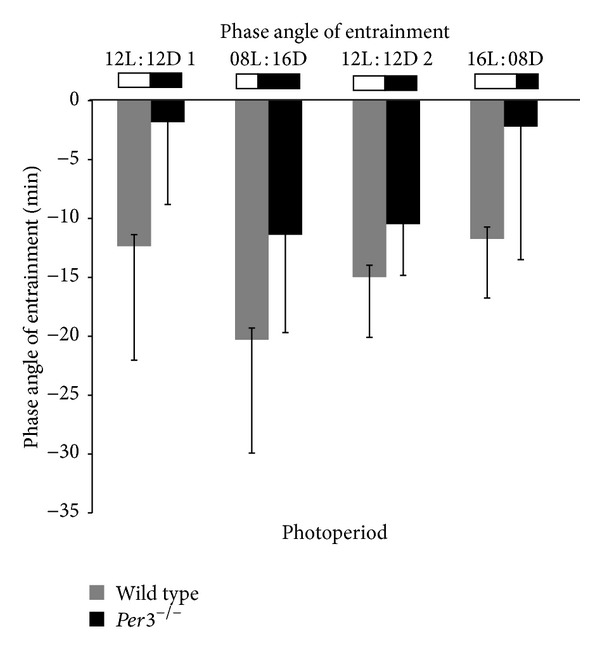
Phase angle of entrainment ofWT and* Per3* ^−*/*−^ mice in all photoperiods analysed. Values are represented as mean ± SD. Gray bars represent wild-type mice, and black bars show* mPer3* ^−*/*−^ mice.

We compared the phase angle of entrainment between WT and* Per3* ^−*/*−^ mice in all photoperiods analysed (Figure 5). No significant difference between groups was observed, but we did notice a tendency to a shorter phase angle in* Per3* ^−*/*−^ mice in 12L : 12D 1 and 16L : 8D (*t* value = −2, 16, *P* = 0.056 and *t* value = −1, 89, *P* = 0.088, resp.).

## 4. Discussion

Based on the actograms and analysis of phase angles of entrainment, we observed that* Per3* ^−*/*−^ mice appeared to need more days to synchronise to the long photoperiod (16L : 8D) than WT mice and show more activity than WT mice in the light phase in 16L : 8D. When mice were released into DD after 16L : 8D, after a few transients in the cases of some animals, the onset of activity shifted to a phase similar to that in the preceding 12L : 12D period, suggesting that the main effect of lacking* Per3* is not a strong direct shift of the phase of the circadian clock (phase of entrainment) but is instead more likely related to a preponderant masking effect of light on the activity behaviour.

In fact, the light signal may be considered a Zeitgeber as well as a masking agent. These roles of light on activity patterns are inseparable during light dark entrainment [14, 15]. Although we did not use a classical protocol to distinguish between masking and entrainment [14], our results are indicative of masking and corroborate a more elaborate protocol applied in our previous work [11]. The fact that the animals, when released into constant darkness after the 16L : 8D cycles, adopted rest-activity rhythms with an onset phase similar to their former 12L : 12D cycle (instead of maintaining the rest-activity profile they displayed in their previous 16L : 8D) supports the interpretation that their activity in 16L : 8D cycles was in fact preponderantly masked.

Studies of* Per3* ^−*/*−^ mice have reported none or only subtle behavioural changes in circadian properties [3, 4, 16–18]. However, these studies used short light pulses as the stimulus. In the present study, the stimulus was chronic light : dark conditions, and the observed behavioural differences between* Per3* ^−*/*−^ and WT mice, especially following long light exposure, suggest that* mPer3* is somehow importantly related to the sensitivity of light.

Thus, it seems that* Per3* is not involved in the processing of acute responses to light, but when animals are exposed to chronic light regimens, changes in behaviour appear. Our results strengthen the hypothesis that* Per3* ^−*/*−^ mice are less sensitive to light and corroborate reports showing that constant light affected the length of the endogenous period of* Per3* ^−*/*−^ differentially compared to WT and that the masking effect of light was attenuated or nonexistent in* mPer3* ^−*/*−^ animals [11].

Studies investigating* Per3 *mRNA expression in suprachiasmatic nuclei are consistent with this; that is, the expression is not responsive to light pulses [16, 17]. However, in animals submitted to different photoperiods, (short photoperiod 10L : 14D and long photoperiod 14L : 10D), changes in* Per3* expression are the most prominent among all clock genes [19].

Our study has some limitations; the number of animals used and the natural individual variability in motor activity limit the power of the analyses and may account for the borderline significance for the phase angle of entrainment in 12L : 12D and 16L : 8D cycles. In addition, we may not exclude an effect of order of the sequence of L : D cycles on behavioural parameters observed.

Studies on humans [6–8], a recent study on* mPer3* ^−*/*−^ mice [11], and the present study indicate that the* Per3 *gene is most likely involved in masking responses and thus may be associated with the interaction between the circadian clock and the motivational drive of behavioural activity in response to light-dark cycle.

## Supplementary Material

Supplementary material shows the periodograms analysis and actograms of all animals included in this study.

## References

[1] Takahashi JS, Hong H-K, Ko CH, McDearmon EL (2008). The genetics of mammalian circadian order and disorder: implications for physiology and disease. *Nature Reviews Genetics*.

[2] von Schantz M (2008). Phenotypic effects of genetic variability in human clock genes on circadian and sleep parameters. *Journal of Genetics*.

[3] Bae K, Jin X, Maywood ES, Hastings MH, Reppert SM, Weaver DR (2001). Differential functions of mPer1, mPer2, and mPer3 in the SCN circadian clock. *Neuron*.

[4] Shearman LP, Jin X, Lee C, Reppert SM, Weaver DR (2000). Targeted disruption of the mPer3 gene: subtle effects on circadian clock function. *Molecular and Cellular Biology*.

[5] Ebisawa T, Uchiyama M, Kajimura N (2001). Association of structural polymorphisms in the human period3 gene with delayed sleep phase syndrome. *EMBO Reports*.

[6] Archer SN, Robilliard DL, Skene DJ (2003). A length polymorphism in the circadian clock gene Per3 is linked to delayed sleep phase syndrome and extreme diurnal preference. *Sleep*.

[7] Pereira DS, Tufik S, Louzada FM (2005). Association of the length polymorphism in the human Per3 gene with the delayed sleep-phase syndrome: does latitude have an influence upon it?. *Sleep*.

[8] Viola AU, Archer SN, James L (2007). PER3 polymorphism predicts sleep structure and waking performance. *Current Biology*.

[9] Hasan S, van der Veen DR, Winsky-Sommerer R, Dijk D-J, Archer SN (2011). Altered sleep and behavioral activity phenotypes in PER3-deficient mice. *American Journal of Physiology—Regulatory Integrative and Comparative Physiology*.

[10] Pendergast JS, Niswender KD, Yamazaki S (2012). Tissue-specific function of period3 in circadian rhythmicity. *PLoS ONE*.

[11] Van der Veen DR, Archer SN (2010). Light-dependent behavioral phenotypes in PER3-deficient mice. *Journal of Biological Rhythms*.

[12] Nadkarni NA, Weale ME, Von Schantz M, Thomas MG (2005). Evolution of a length polymorphism in the human PER3 gene, a component of the circadian system. *Journal of Biological Rhythms*.

[13] Sokolove PG, Bushell WN (1978). The chi square periodogram: its utility for analysis of circadian rhythms. *Journal of Theoretical Biology*.

[14] Redlin U, Mrosovsky N (1999). Masking of locomotor activity in hamsters. *Journal of Comparative Physiology A*.

[15] Marques MD, Waterhouse JM (1994). Masking and the evolution of circadian rhythmicity. *Chronobiology International*.

[16] Zylka MJ, Shearman LP, Weaver DR, Reppert SM (1998). Three period homologs in mammals: differential light responses in the suprachiasmatic circadian clock and oscillating transcripts outside of brain. *Neuron*.

[17] Takumi T, Taguchi K, Miyake S (1998). A light-independent oscillatory gene mPer3 in mouse SCN and OVLT. *The EMBO Journal*.

[18] Pendergast JS, Friday RC, Yamazaki S (2010). Photic entrainment of period mutant mice is predicted from their phase response curves. *Journal of Neuroscience*.

[19] Tournier BB, Menet JS, Dardente H (2003). Photoperiod differentially regulates clock genes’ expression in the suprachiasmatic nucleus of Syrian hamster. *Neuroscience*.

