# Supplementary material for: The Effect of Different Photoperiods in Circadian Rhythms of Per3 Knockout Mice
Source: Biomed Res Int. 2014 May 8;2014:170795. doi: 10.1155/2014/170795 (PMC4042019; doi:10.1155/2014/170795)
Supplement: Supplementary file 1 — Supplementary material shows the periodograms analysis and actograms of all animals included in this study. [file 170795.f1.pdf]

Supplemental Material

Periodograms were constructed for each animal in all light conditions. The animals are labelled 1-12. All periodograms and double plotted actograms of running wheel activity are shown below, together with the number of the corresponding animal.

Animal 1.

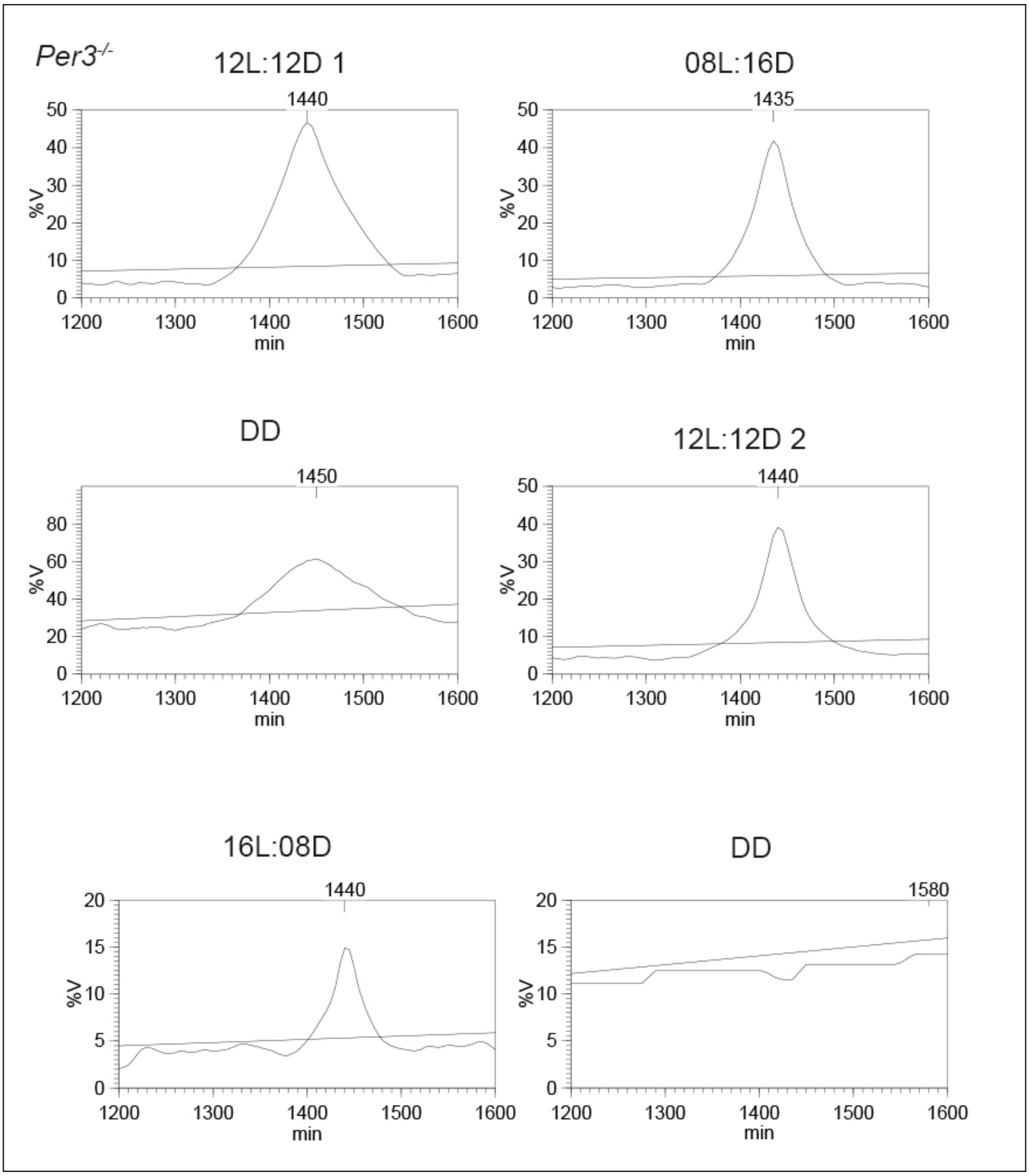

Animal 1

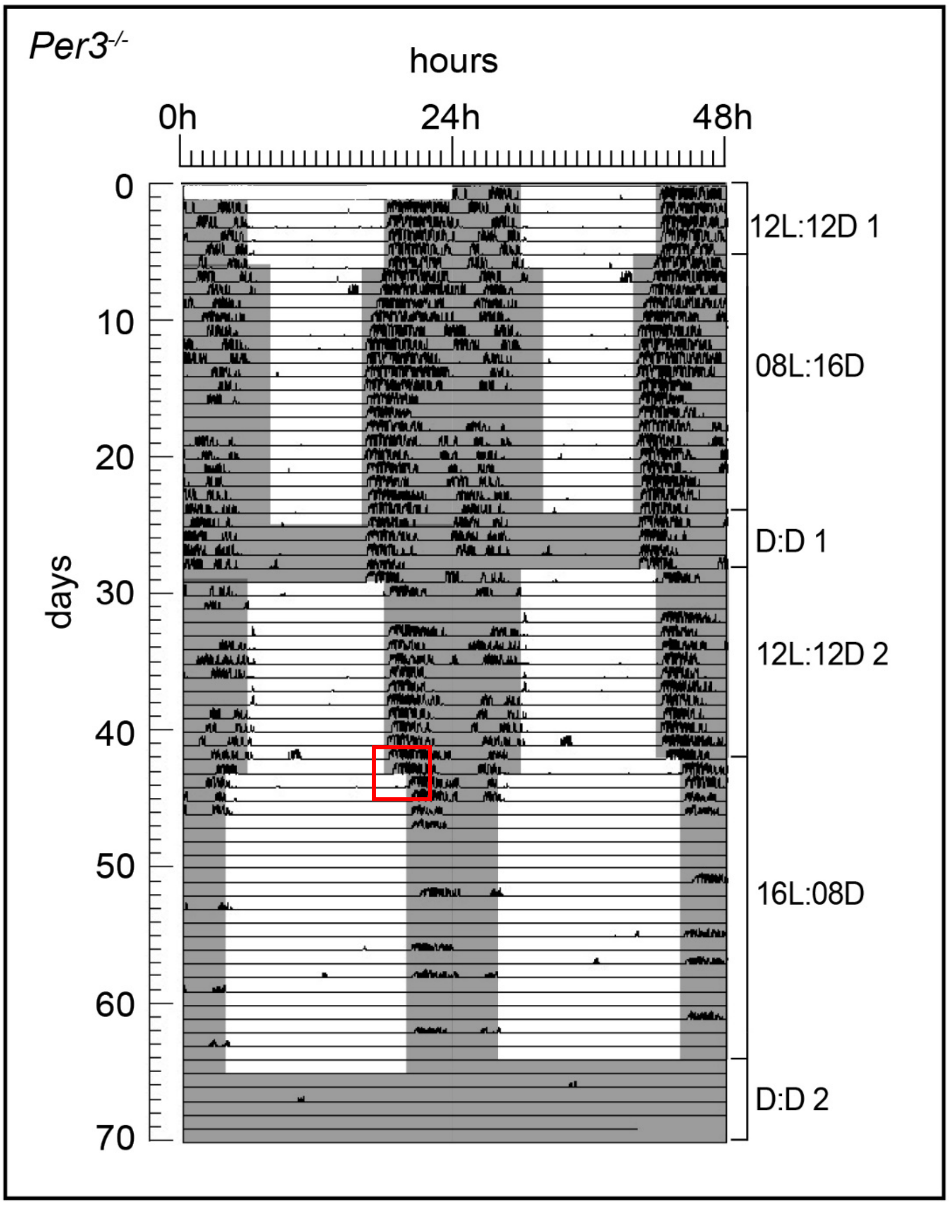

Per3-/-
hours
0h
24h
48h
days
0
10
20
30
40
50
60
70
12L:12D 1
08L:16D
D:D 1
12L:12D 2
16L:08D
D:D 2

Animal 2.

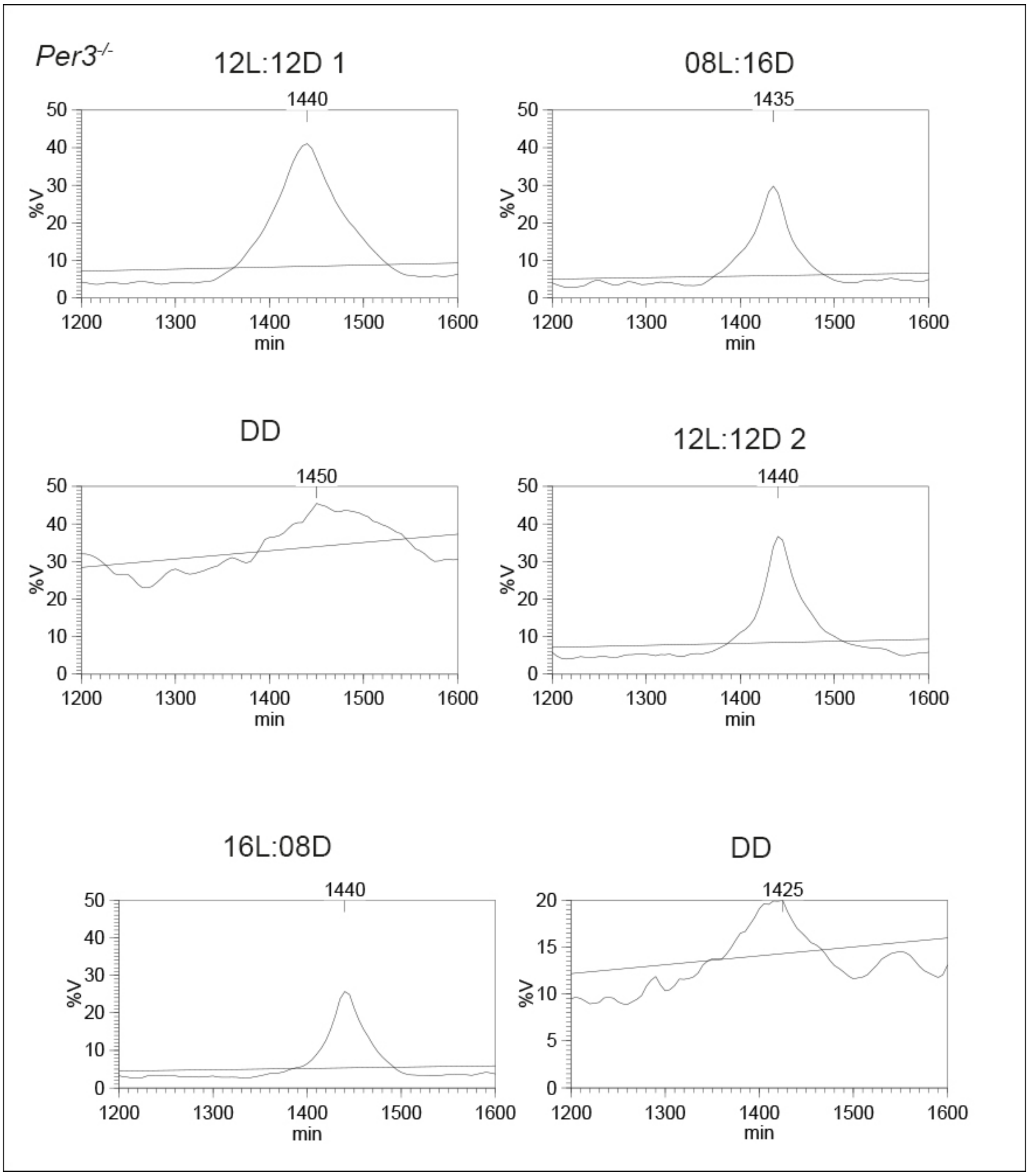

Per3-/-
12L:12D 1
1440
08L:16D
1435
DD
1450
12L:12D 2
1440
16L:08D
1440
DD
1425
%V
min

Animal 2

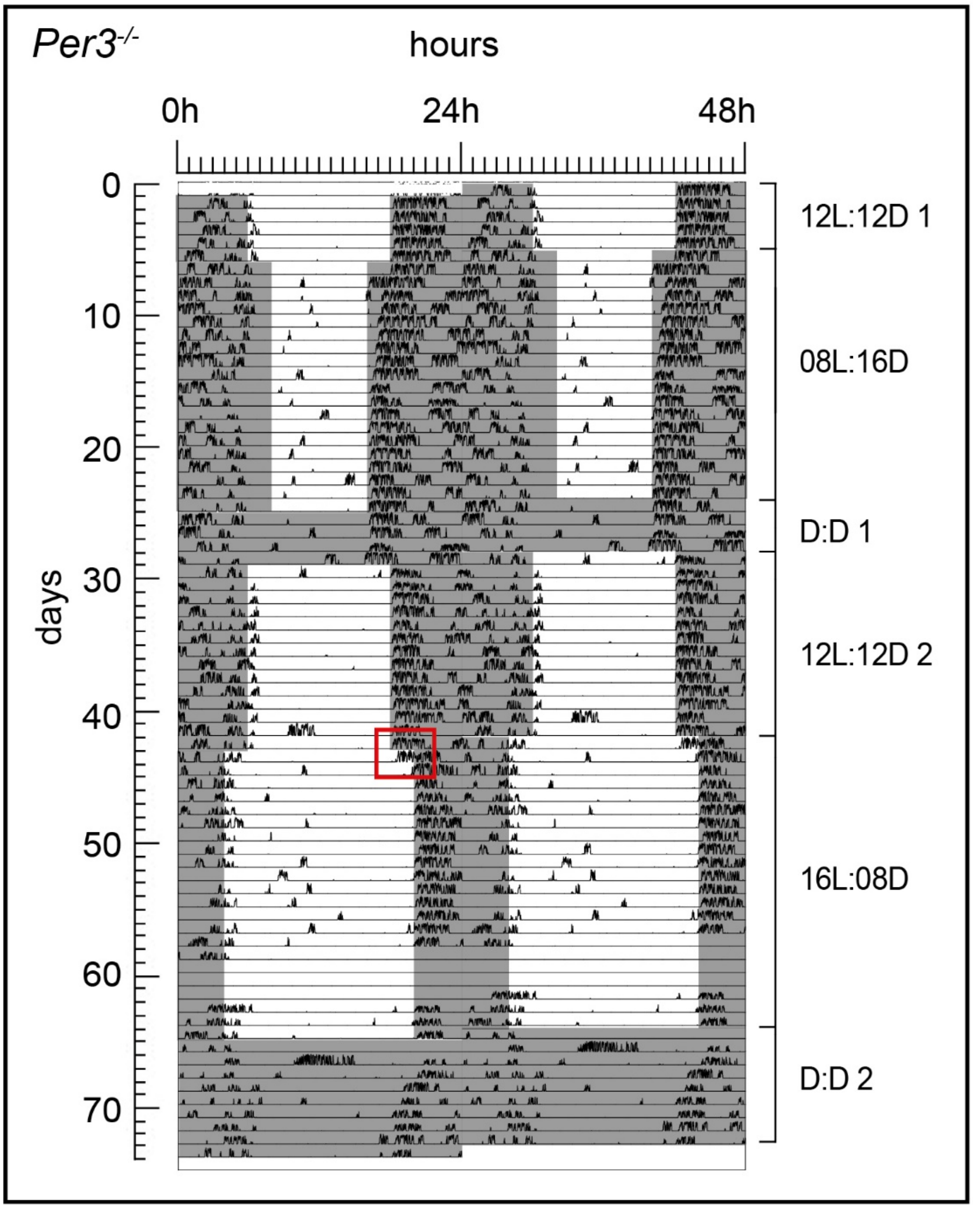
Per3-/-
hours
0h
24h
48h
days
0
10
20
30
40
50
60
70
12L:12D 1
08L:16D
D:D 1
12L:12D 2
16L:08D
D:D 2

Animal 3

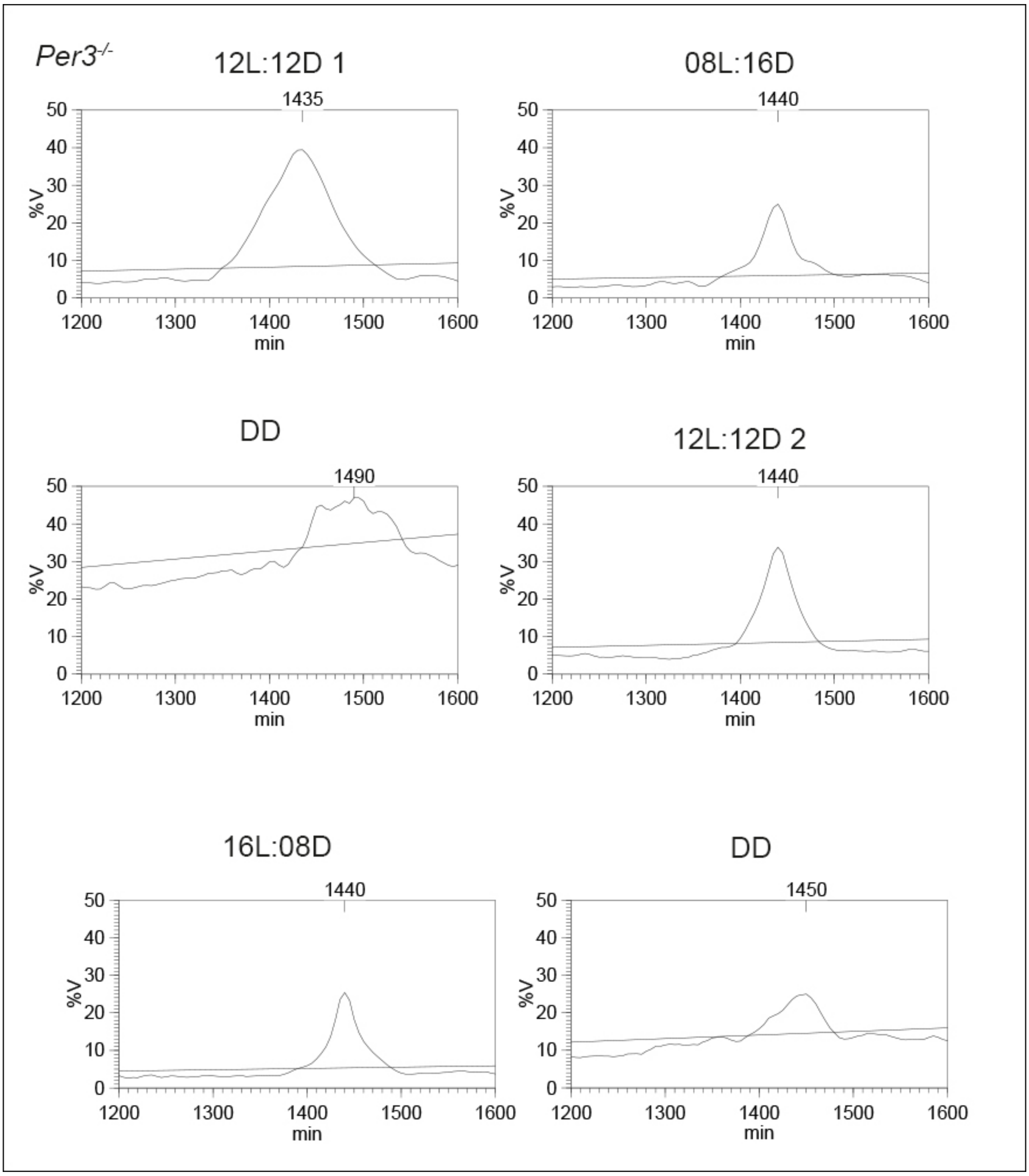

Per3-/-
12L:12D 1
1435
08L:16D
1440
DD
1490
12L:12D 2
1440
16L:08D
1440
DD
1450
%V
min
50
40
30
20
10
0
1200
1300
1400
1500
1600

Animal 3

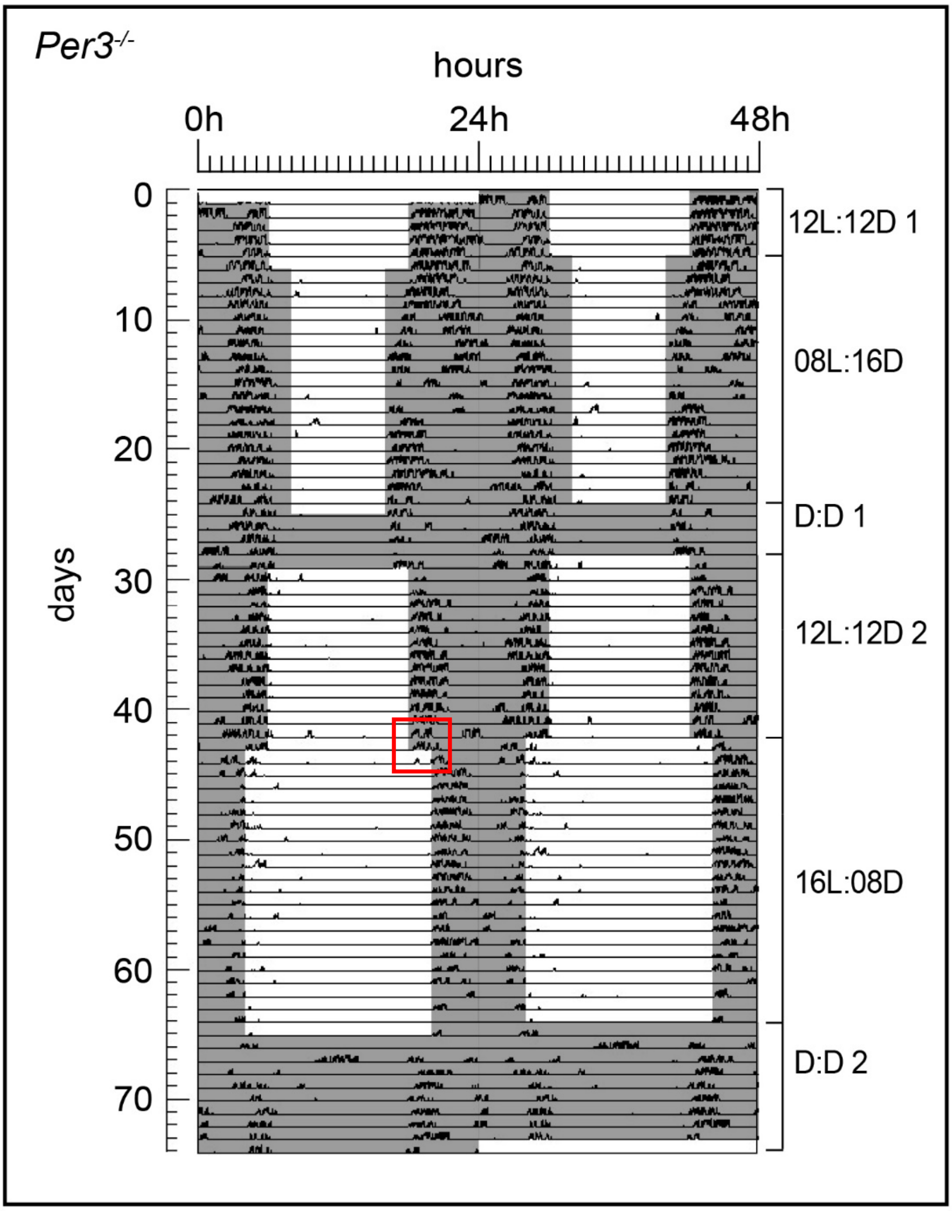

Per3-/-
hours
0h
24h
48h
days
0
10
20
30
40
50
60
70
12L:12D 1
08L:16D
D:D 1
12L:12D 2
16L:08D
D:D 2

Animal 4.

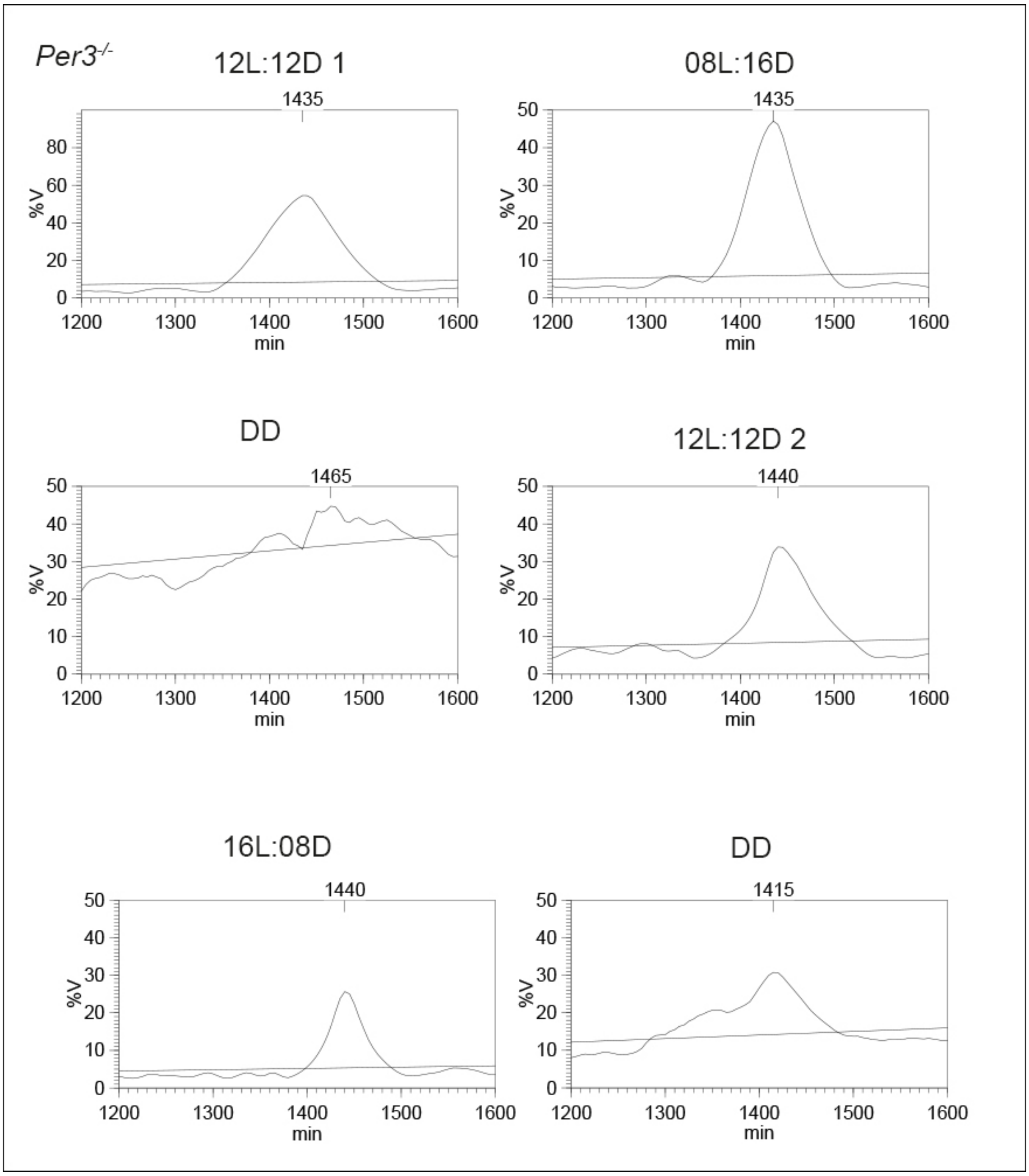

Per3$^{-/-}$
12L:12D 1
1435
%V
min
08L:16D
1435
DD
1465
12L:12D 2
1440
16L:08D
1440
DD
1415

Animal 4

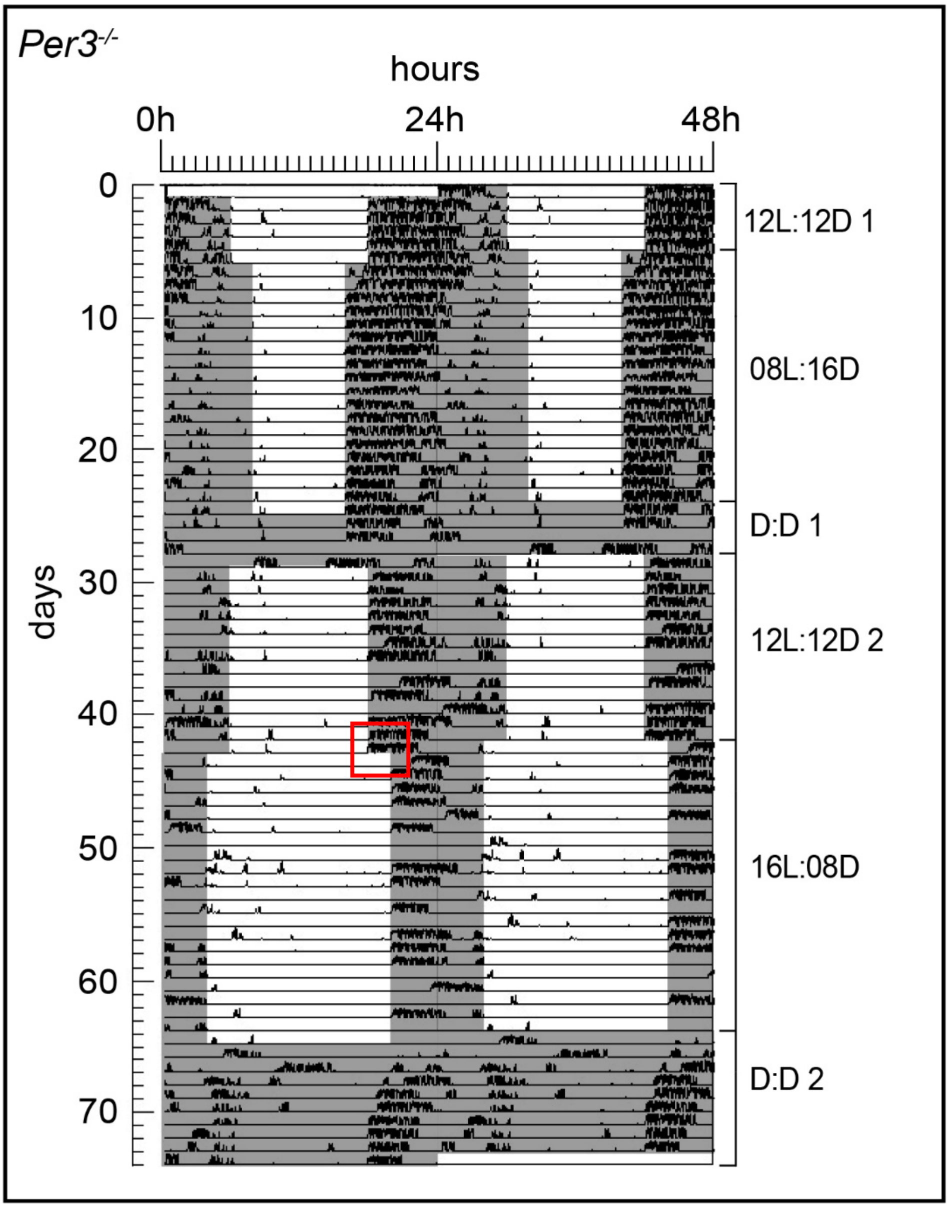

Per3-/-
hours
0h
24h
48h
days
0
10
20
30
40
50
60
70
12L:12D 1
08L:16D
D:D 1
12L:12D 2
16L:08D
D:D 2

Animal 5

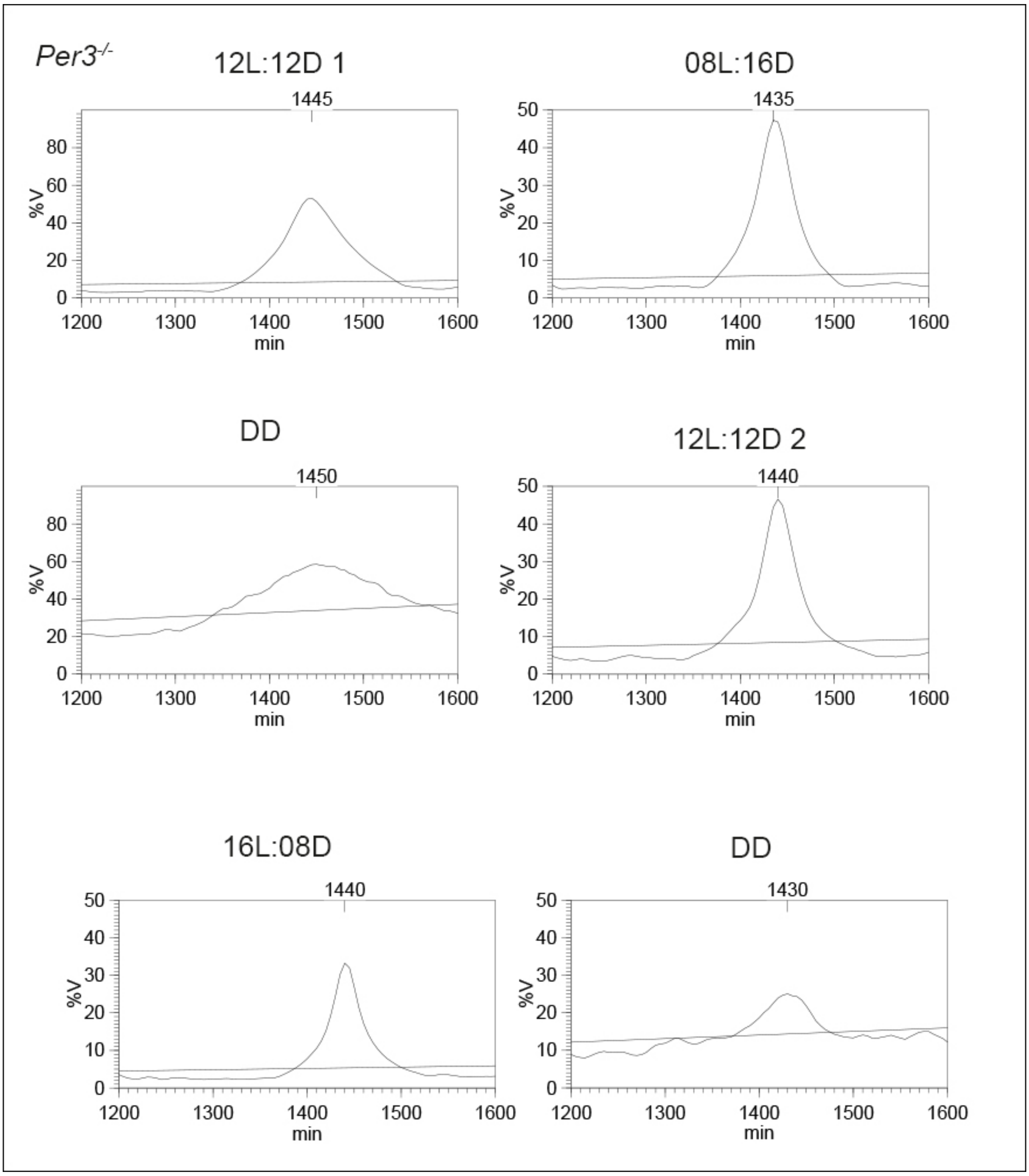

Per3-/-
12L:12D 1
1445
80
60
40
20
0
%V
1200
1300
1400
1500
1600
min
08L:16D
1435
50
40
30
20
10
0
DD
1450
12L:12D 2
1440
16L:08D
1440
DD
1430

Animal 5

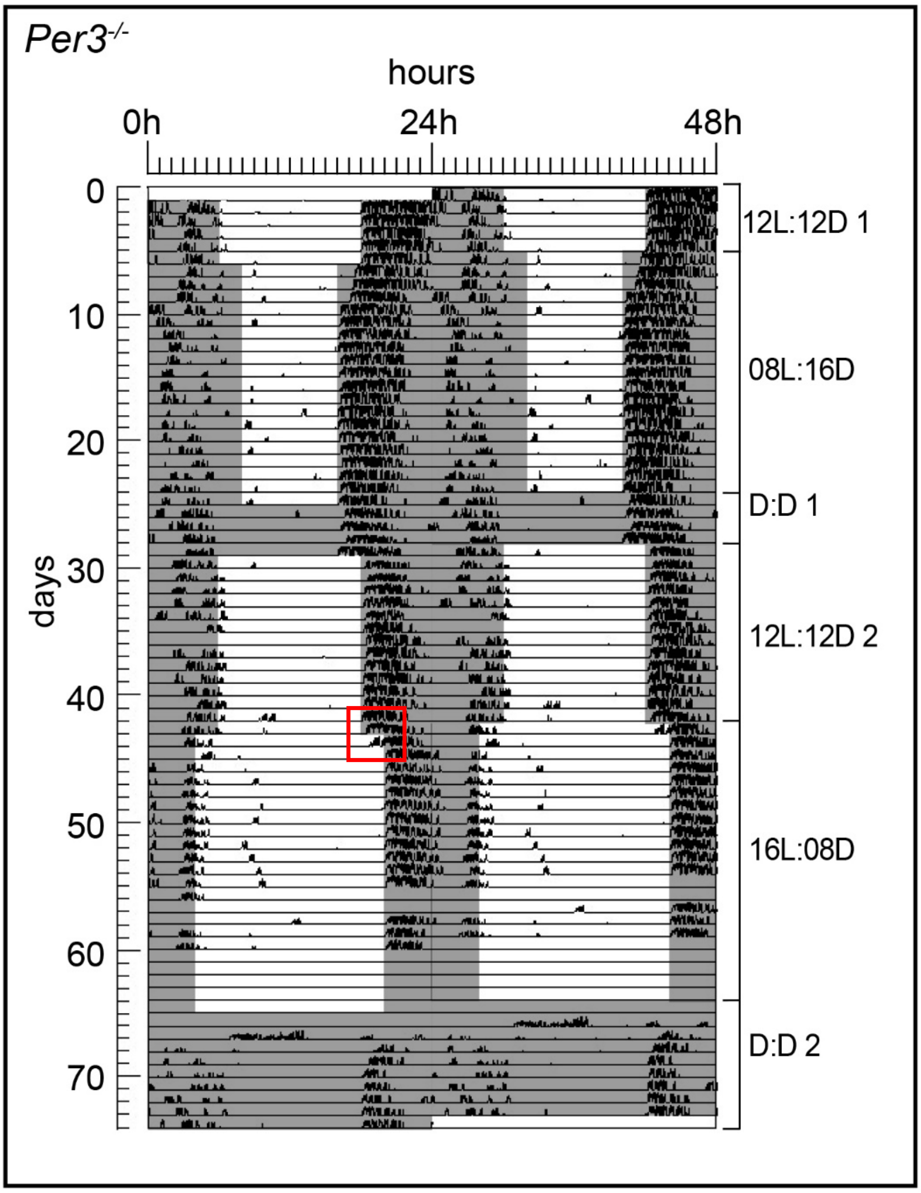

Per3-/-
hours
0h
24h
48h
days
0
10
20
30
40
50
60
70
12L:12D 1
08L:16D
D:D 1
12L:12D 2
16L:08D
D:D 2

Animal 6.

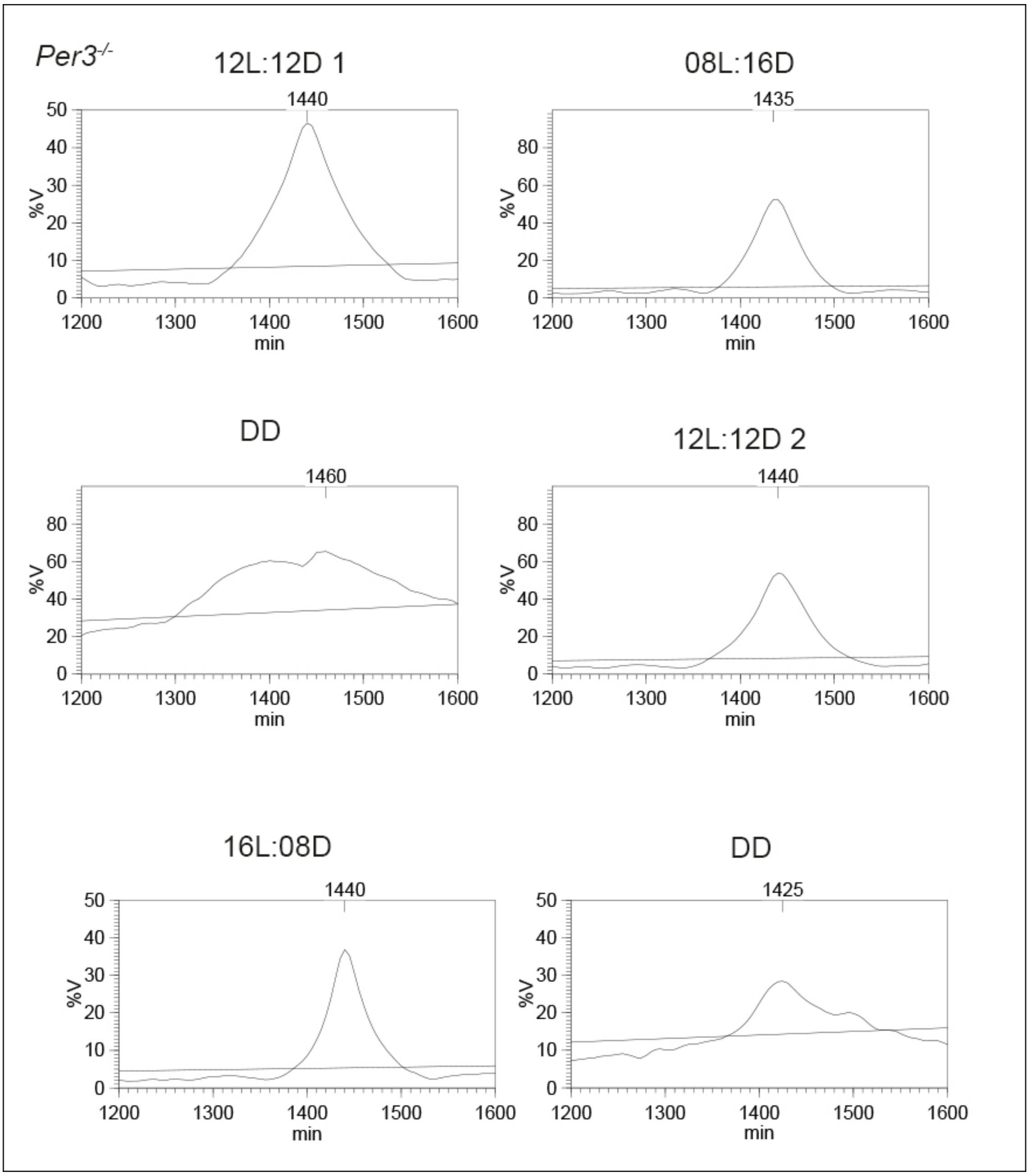

Per3-/-
12L:12D 1
08L:16D
DD
12L:12D 2
16L:08D
DD
1440
1435
1460
1440
1440
1425
%V
min

Animal 6

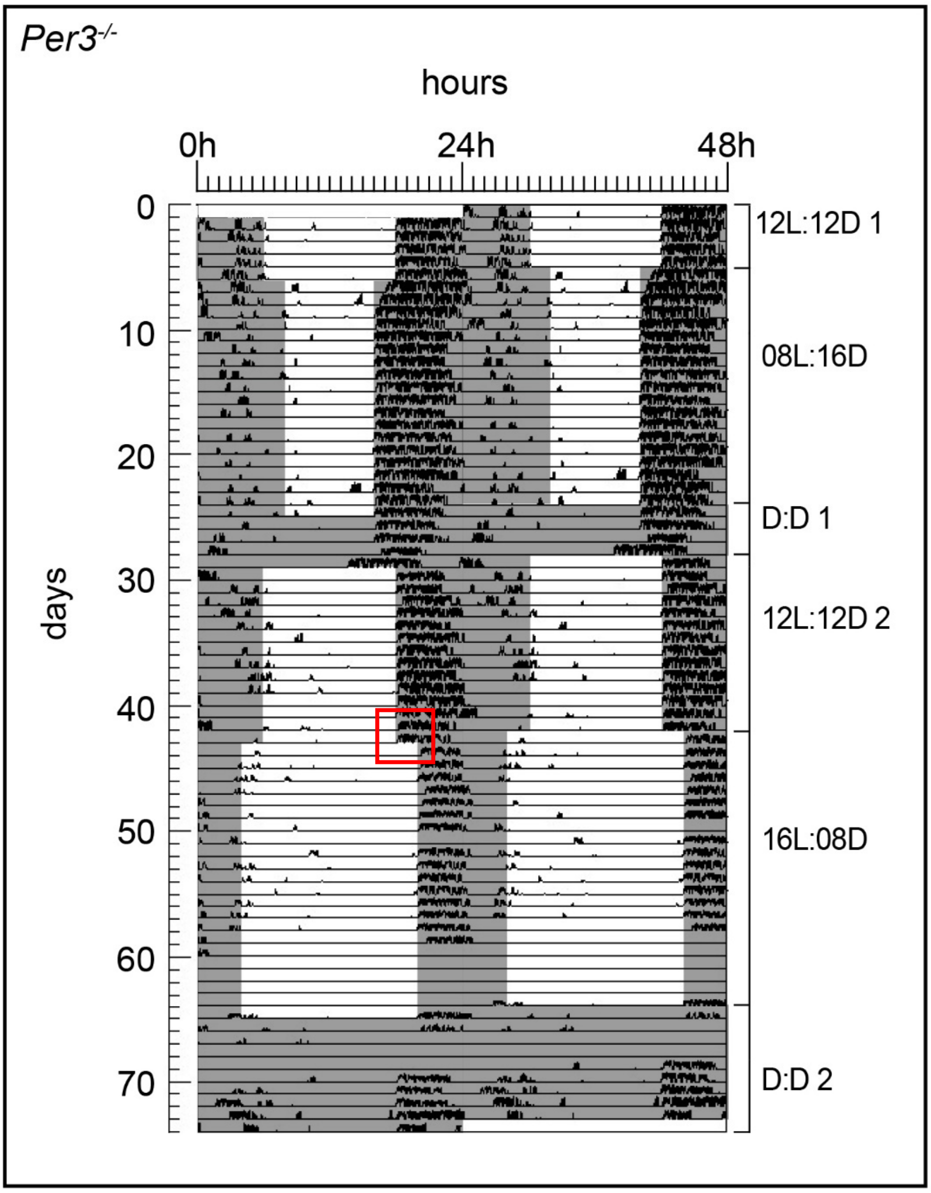

Per3-/-
hours
0h
24h
48h
days
0
10
20
30
40
50
60
70
12L:12D 1
08L:16D
D:D 1
12L:12D 2
16L:08D
D:D 2

Animal 7.

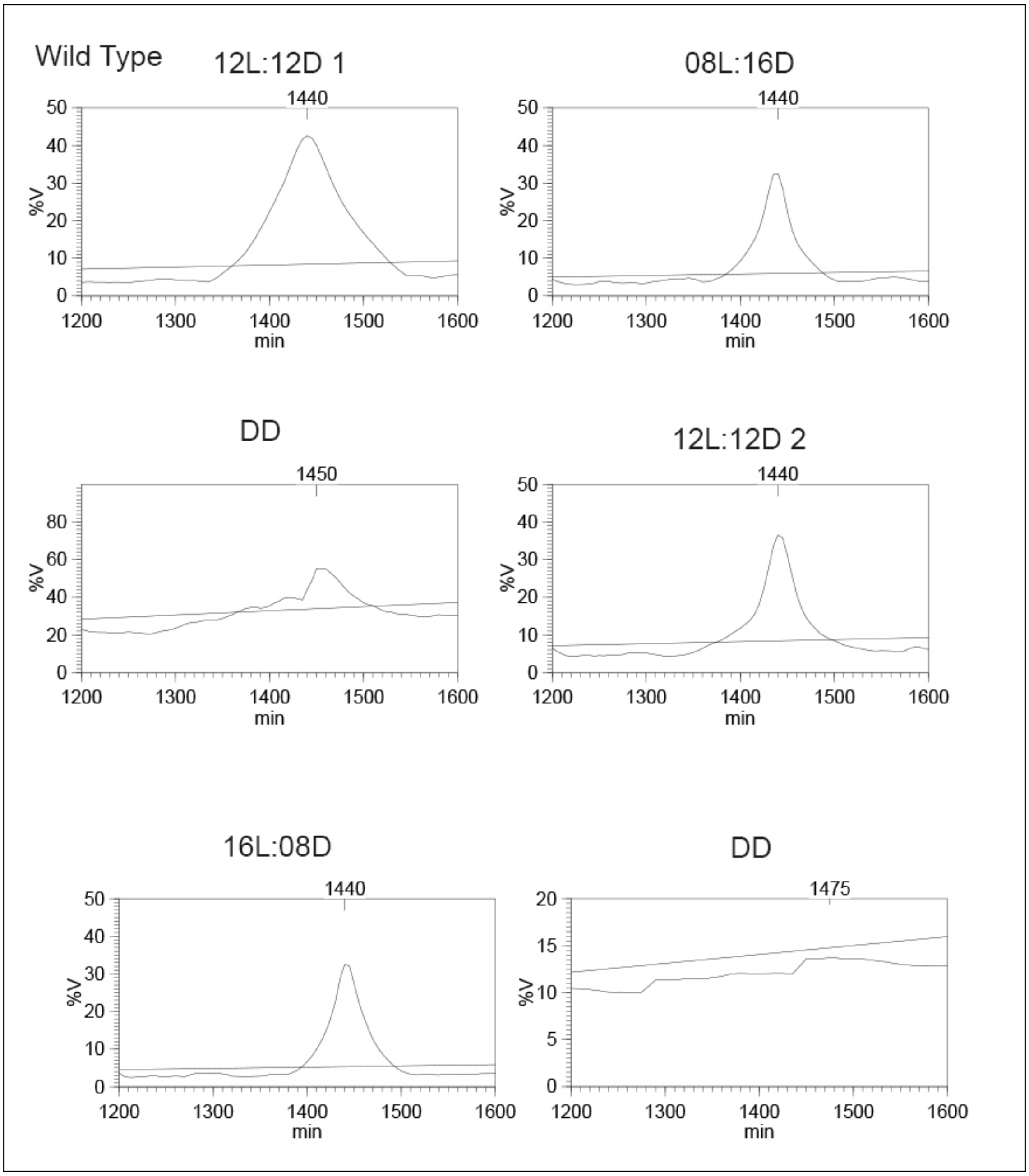
Wild Type
12L:12D 1
1440
08L:16D
1440
DD
1450
12L:12D 2
1440
16L:08D
1440
DD
1475
%V
min

Animal 7

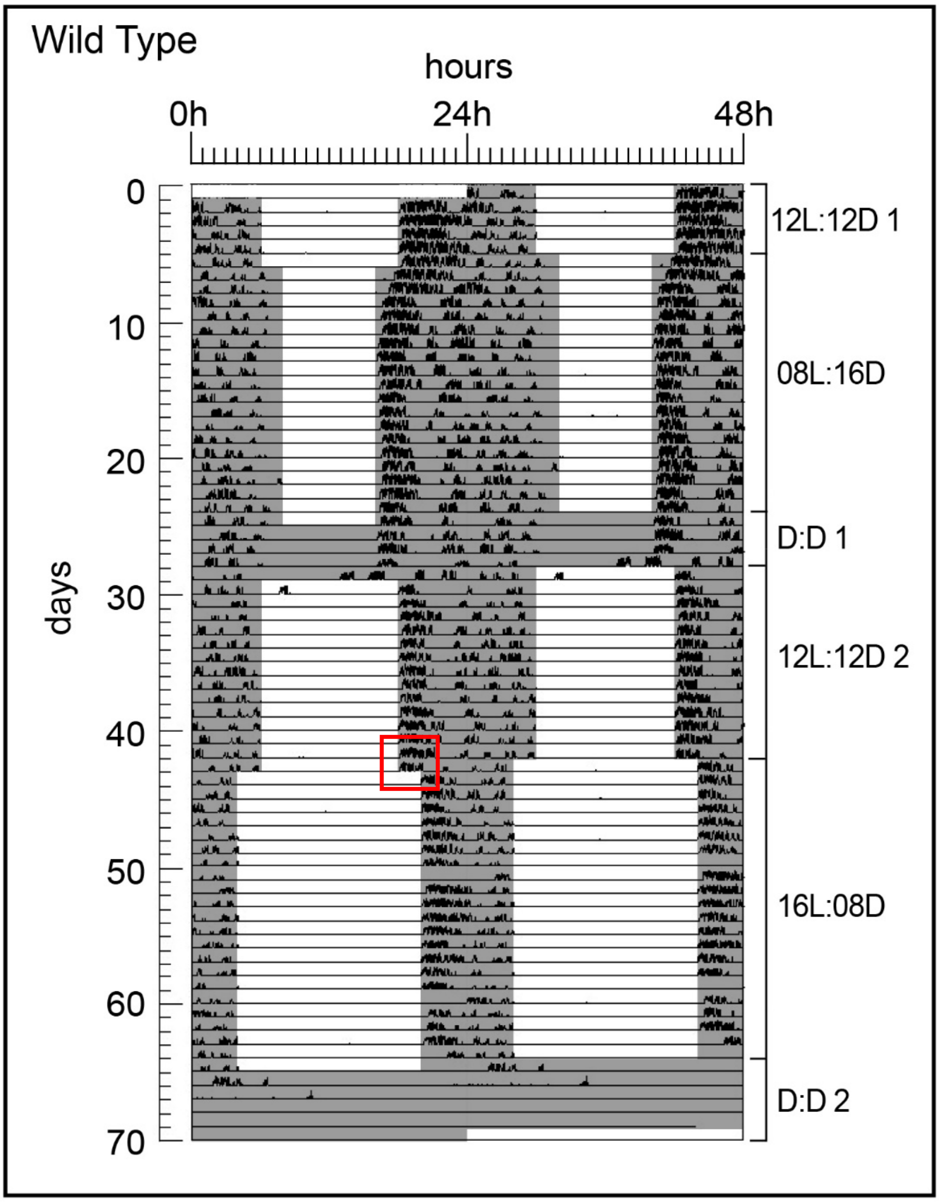

Wild Type
hours
0h
24h
48h
0
10
20
30
40
50
60
70
days
12L:12D 1
08L:16D
D:D 1
12L:12D 2
16L:08D
D:D 2

Animal 8.

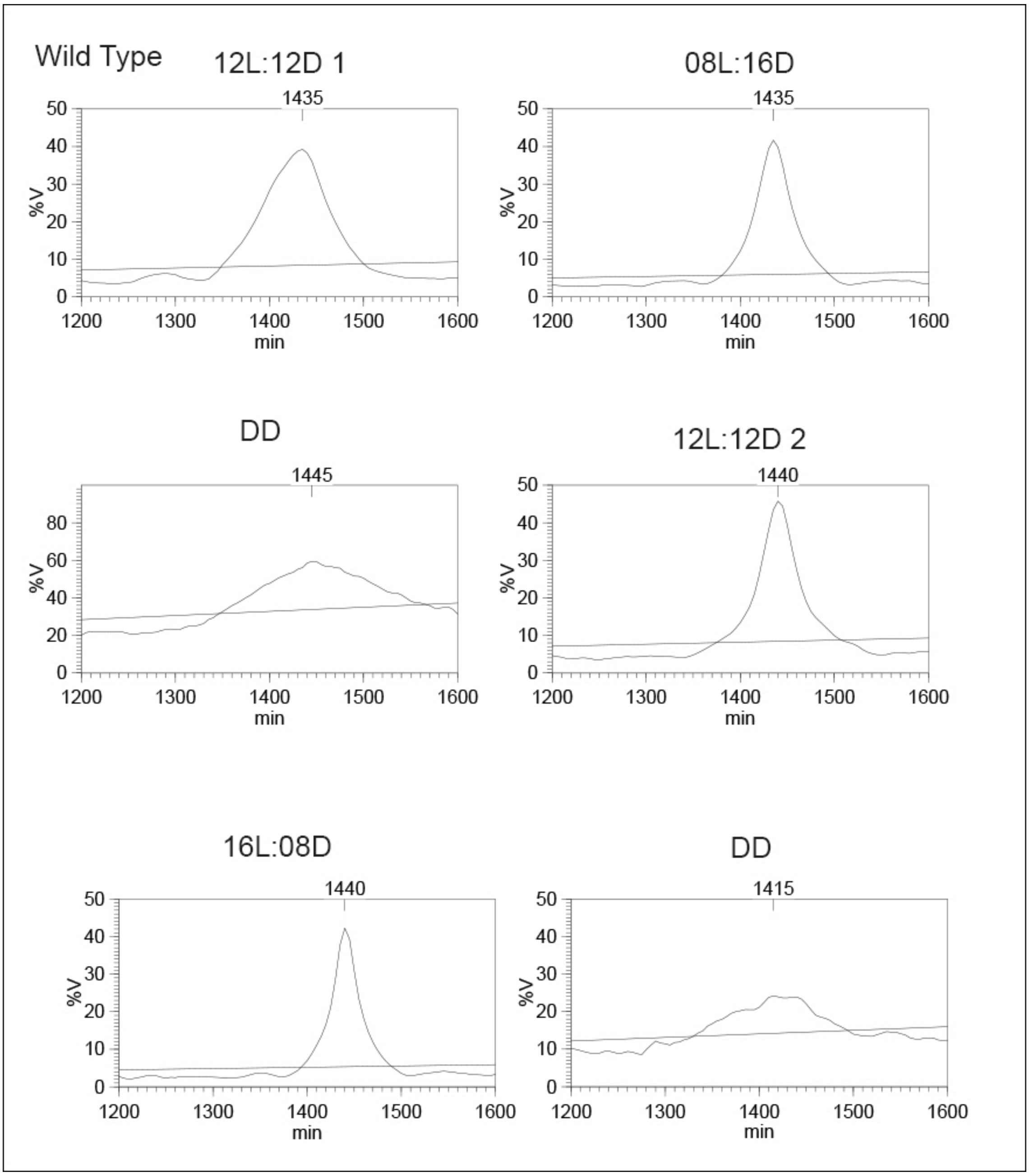
Wild Type
12L:12D 1
1435
08L:16D
1435
DD
1445
12L:12D 2
1440
16L:08D
1440
DD
1415
%V
min

Animal 8

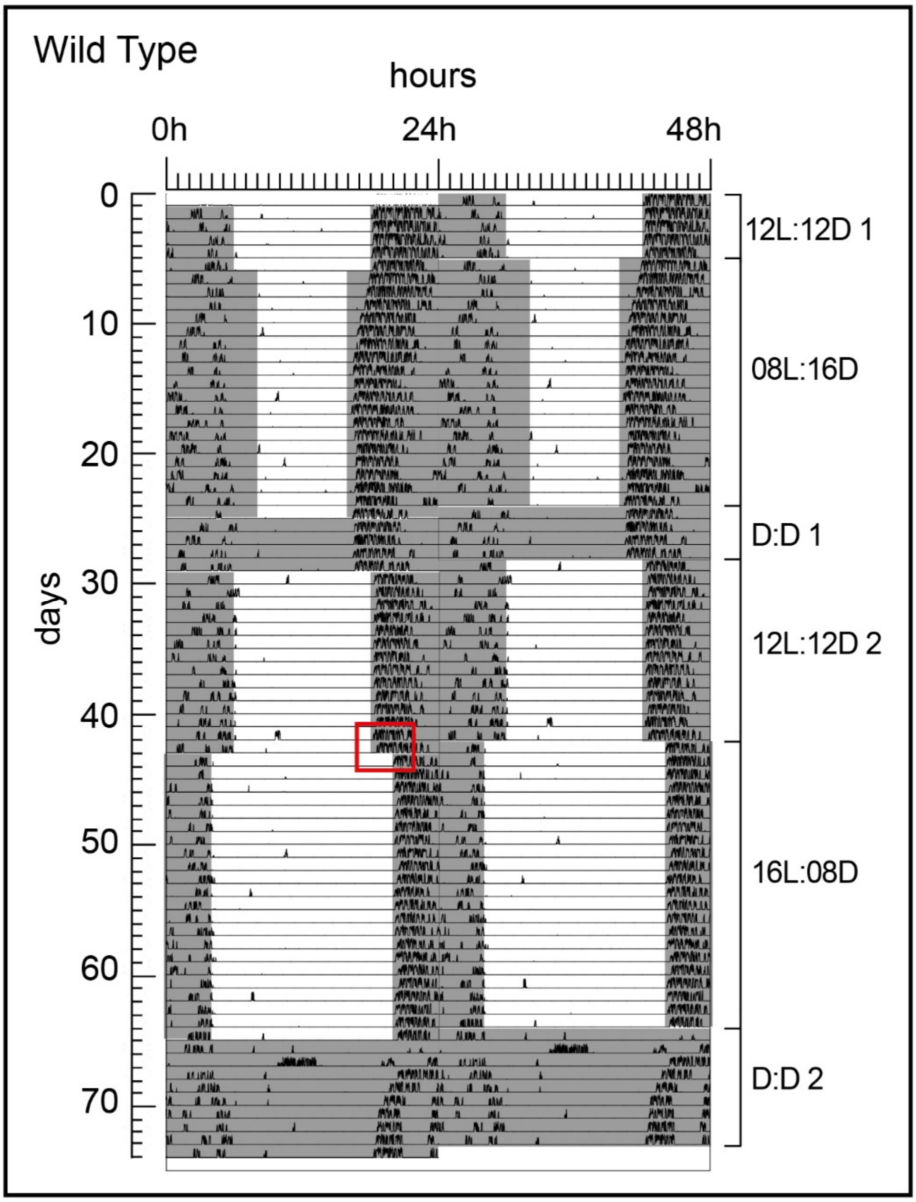

Wild Type
hours
0h
24h
48h
days
0
10
20
30
40
50
60
70
12L:12D 1
08L:16D
D:D 1
12L:12D 2
16L:08D
D:D 2

Animal 9.

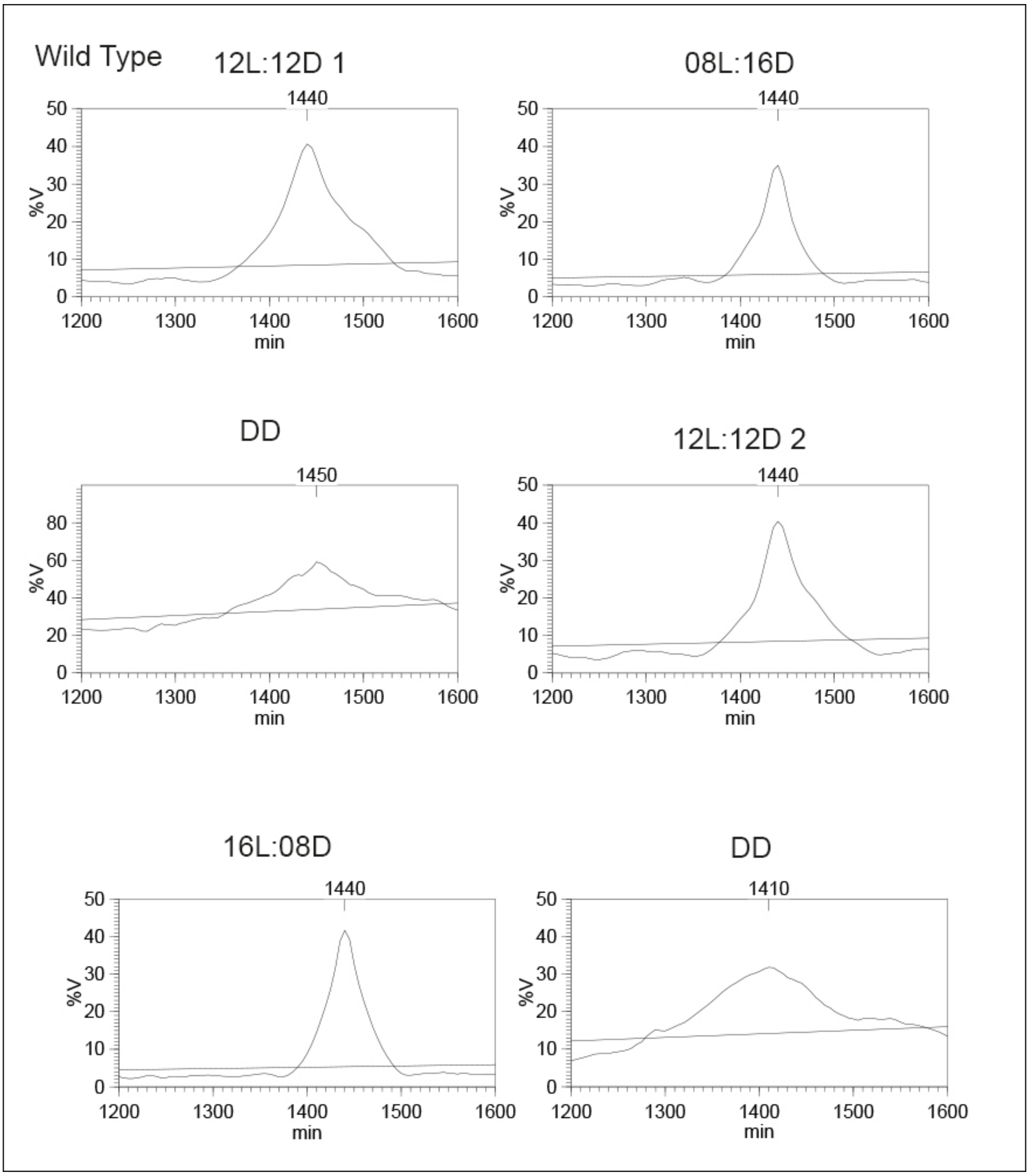

Wild Type
12L:12D 1
1440
08L:16D
1440
DD
1450
12L:12D 2
1440
16L:08D
1440
DD
1410
%V
min
0
10
20
30
40
50
60
80
1200
1300
1400
1500
1600

Animal 9

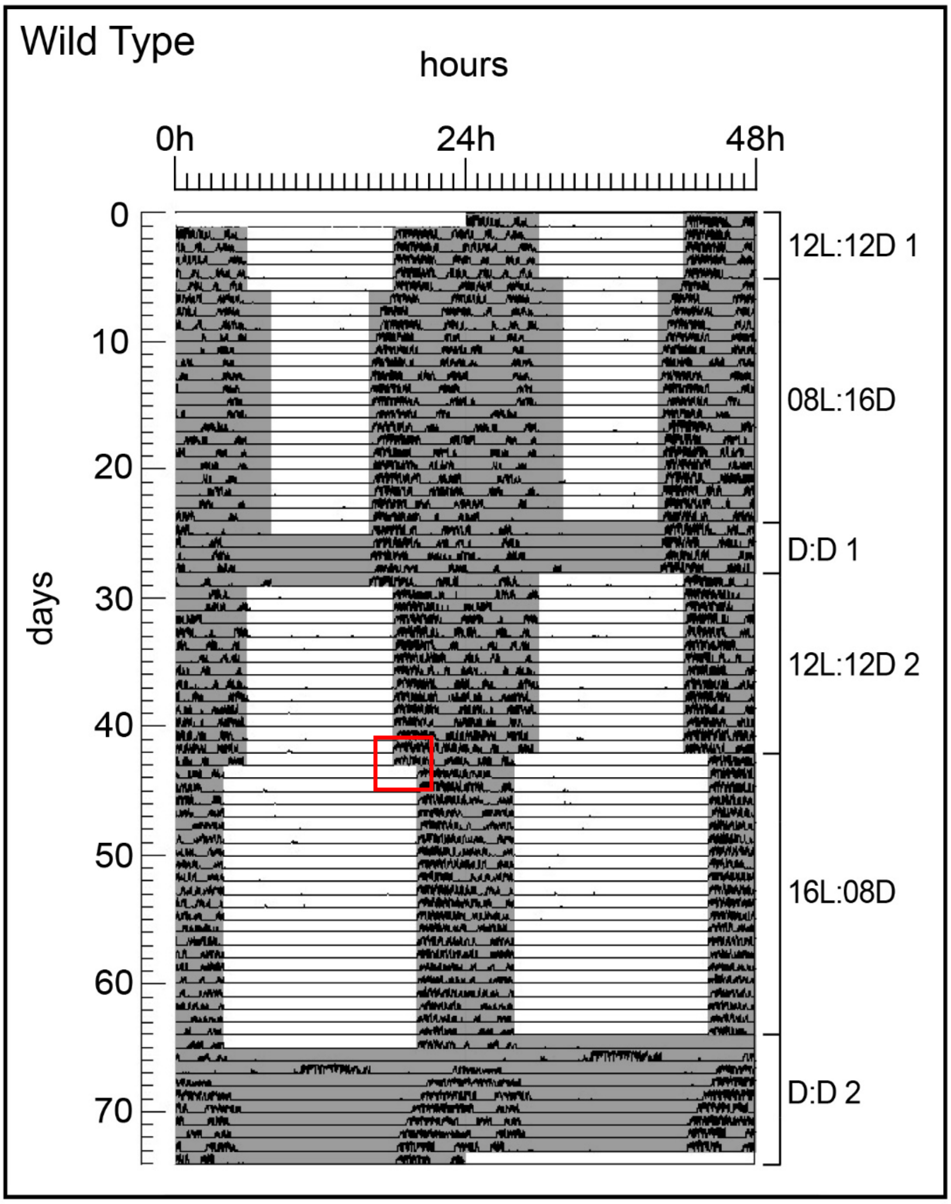
Wild Type
hours
0h
24h
48h
days
0
10
20
30
40
50
60
70
12L:12D 1
08L:16D
D:D 1
12L:12D 2
16L:08D
D:D 2

Animal 10.

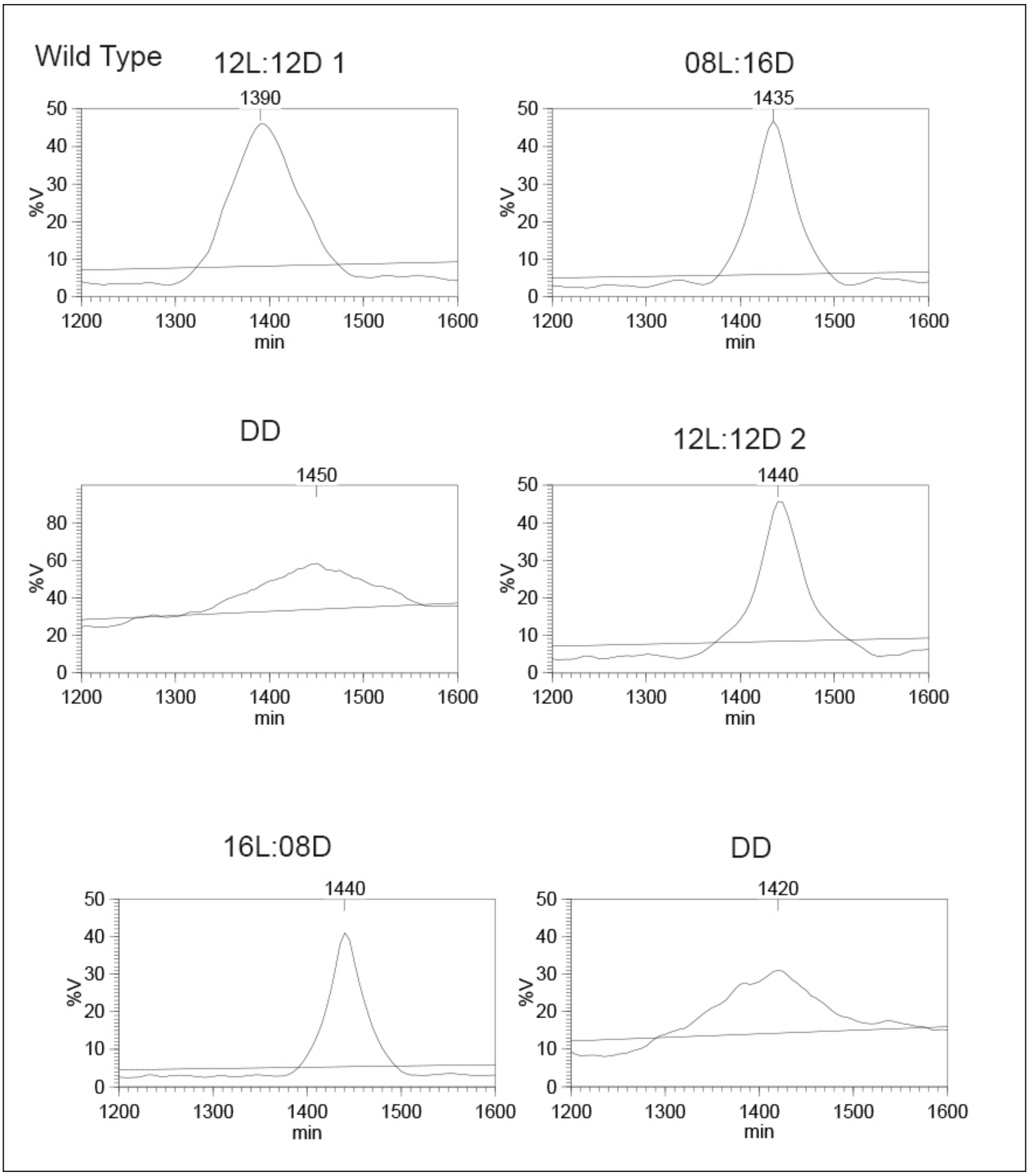
Wild Type
12L:12D 1
1390
08L:16D
1435
DD
1450
12L:12D 2
1440
16L:08D
1440
DD
1420
%V
min

Animal 10

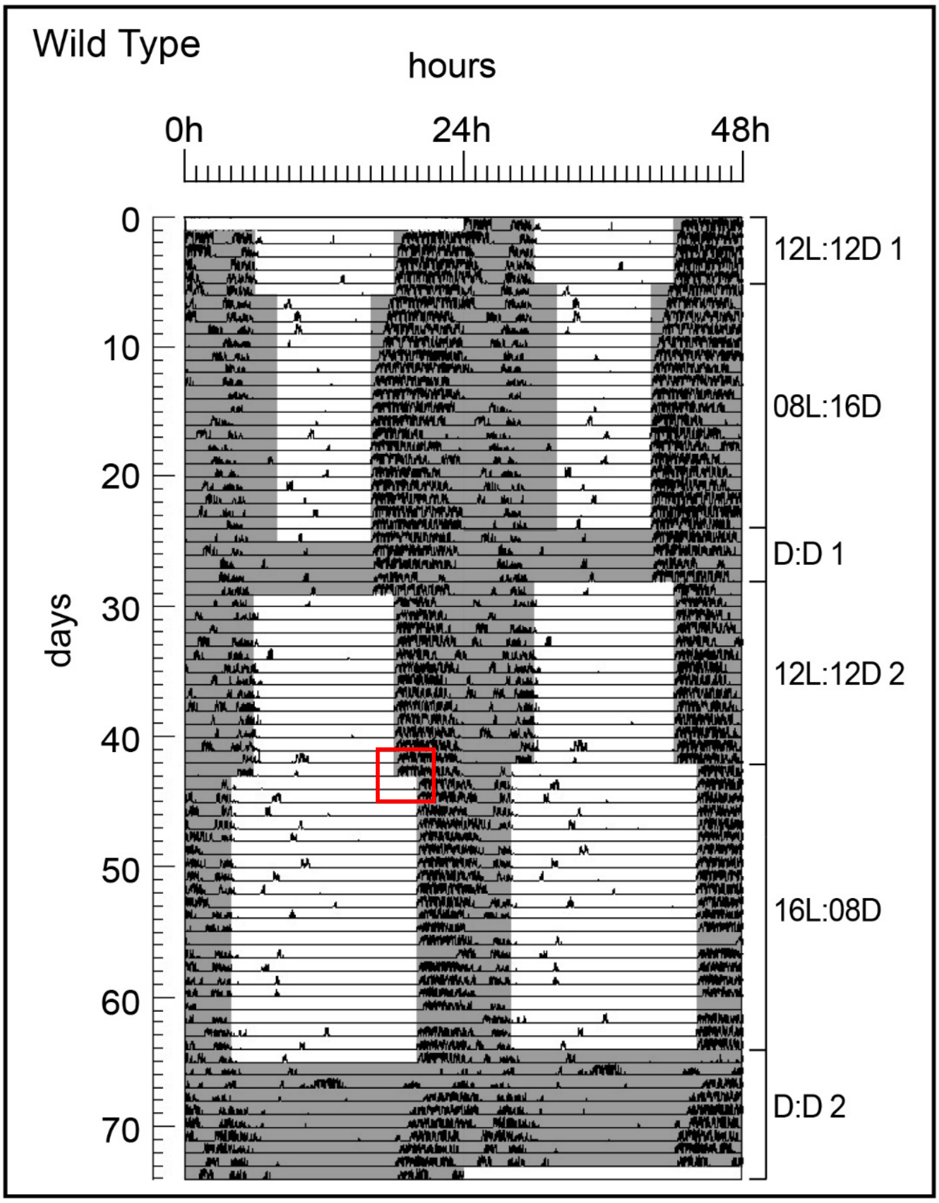

Wild Type
hours
0h
24h
48h
days
0
10
20
30
40
50
60
70
12L:12D 1
08L:16D
D:D 1
12L:12D 2
16L:08D
D:D 2

Animal 11.

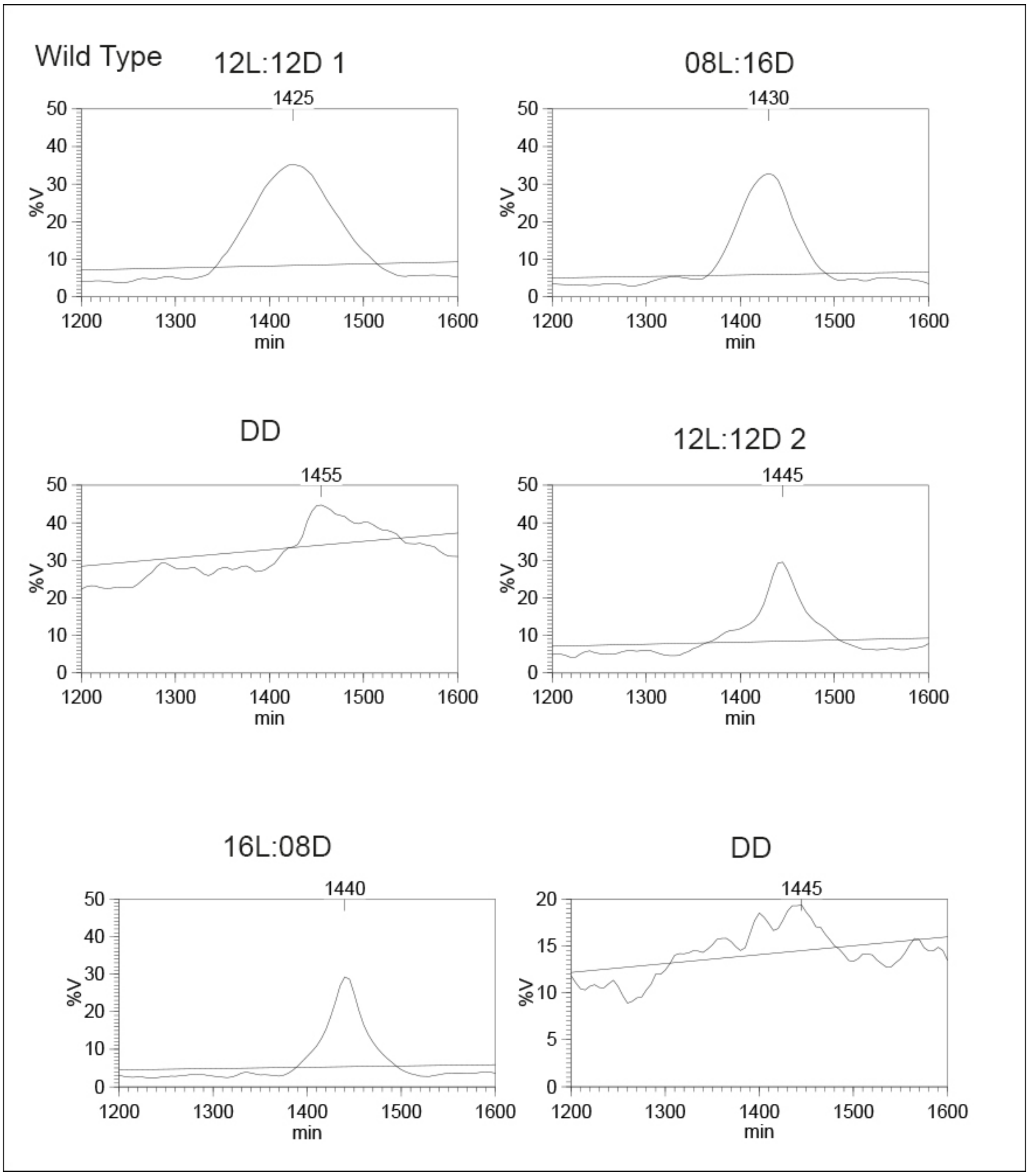
Wild Type
12L:12D 1
1425
08L:16D
1430
DD
1455
12L:12D 2
1445
16L:08D
1440
DD
1445
%V
min

Animal 11

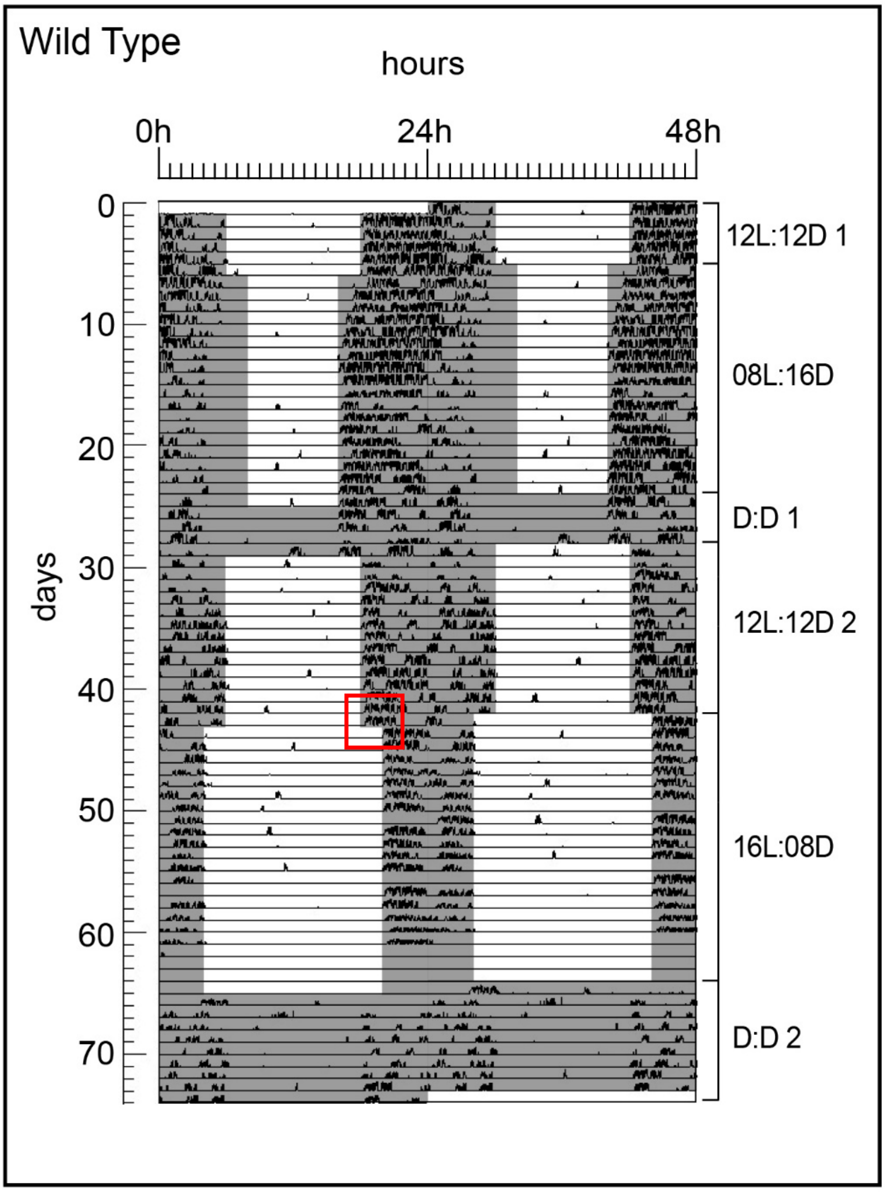
Wild Type
hours
0h
24h
48h
days
0
10
20
30
40
50
60
70
12L:12D 1
08L:16D
D:D 1
12L:12D 2
16L:08D
D:D 2

Animal 12.

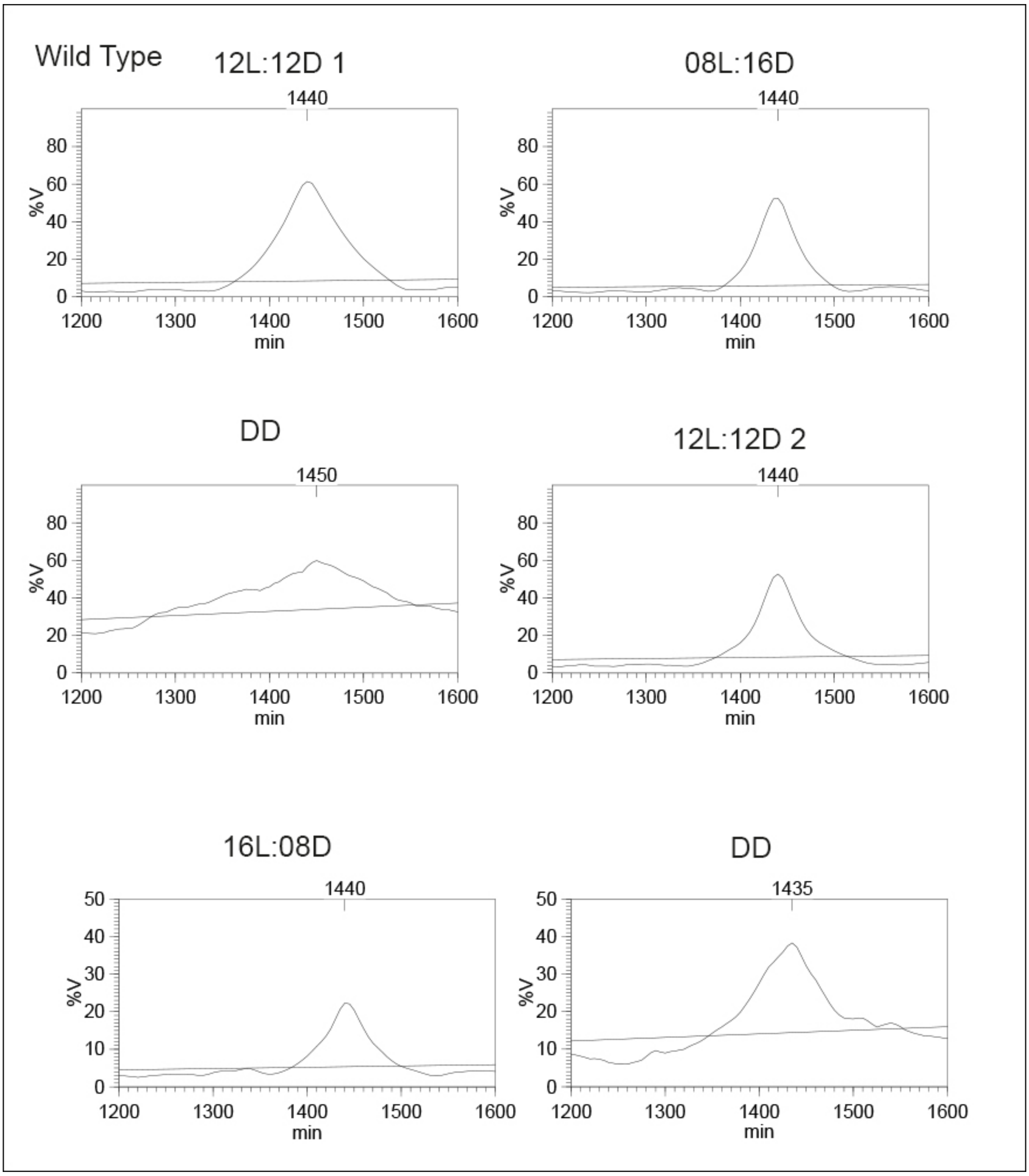

Wild Type
12L:12D 1
08L:16D
DD
12L:12D 2
16L:08D
DD
1440
1440
1450
1440
1440
1435
%V
min

Animal 12

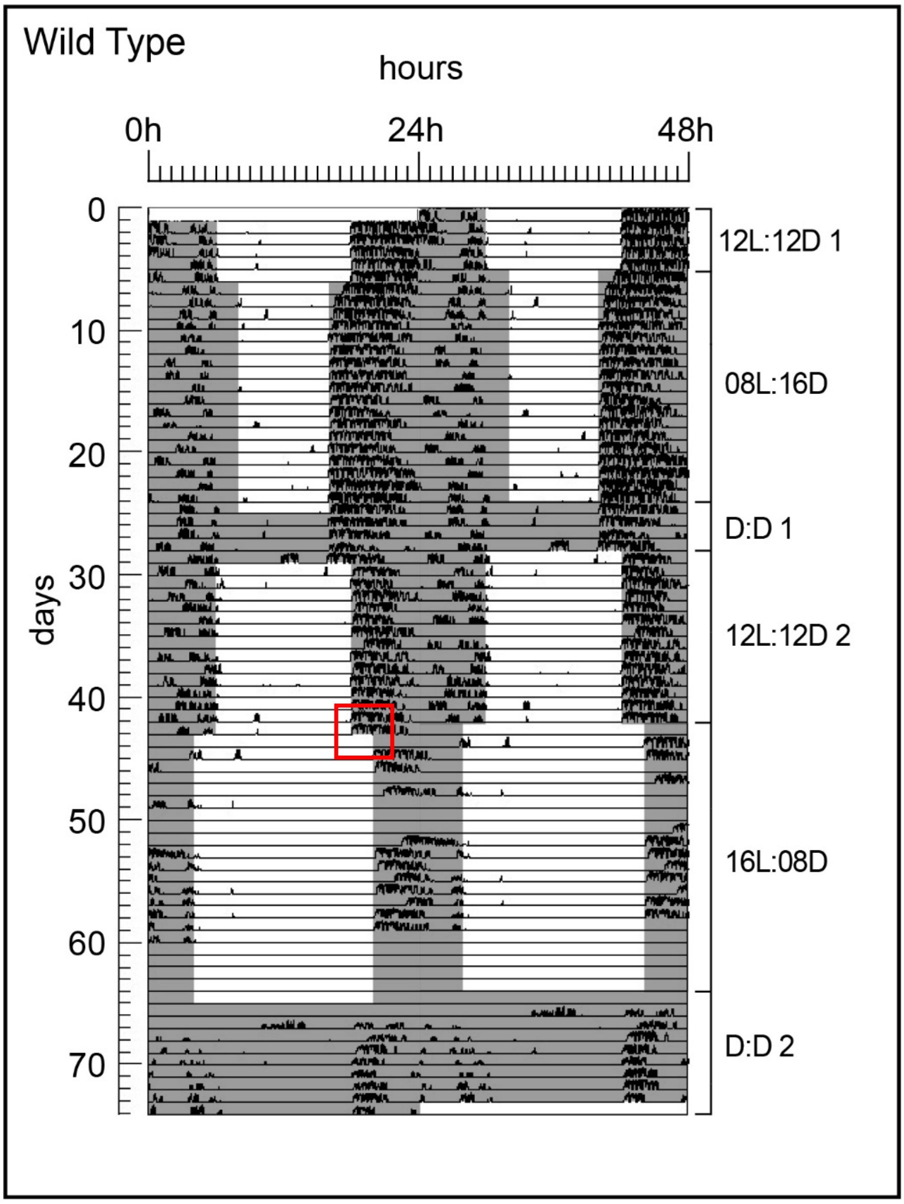

Wild Type
hours
0h
24h
48h
0
10
20
30
40
50
60
70
days
12L:12D 1
08L:16D
D:D 1
12L:12D 2
16L:08D
D:D 2
